# Supplementary material for: Mendelian randomisation for mediation analysis: current methods and challenges for implementation
Source: Eur J Epidemiol. 2021 May 7;36(5):465–78. doi: 10.1007/s10654-021-00757-1 (PMC8159796; doi:10.1007/s10654-021-00757-1)
Supplement: Supplementary file 1 — Supplementary file1 (DOCX 48532 kb) [file 10654_2021_757_MOESM1_ESM.docx]

# Mendelian randomisation for mediation analysis: current methods and challenges for implementation

**Electronic Supplementary Material 1: Supplementary results and figures from simulation analyses**

Alice R Carter ^1,2^*, Eleanor Sanderson ^1,2^, Gemma Hammerton ^1,2,3^, Rebecca C Richmond ^1,2^, George Davey Smith ^1,2,4^, Jon Heron ^1,2,3^, Amy E Taylor ^1,2,4^, Neil M Davies ^1,2,5^, Laura D Howe ^1,2^

1. MRC Integrative Epidemiology Unit, University of Bristol, Bristol, UK
2. Population Health Sciences, Bristol Medical School, University of Bristol, Bristol, UK
3. Centre for Academic Mental Health, University of Bristol, Bristol, UK
4. National Institute for Health Research Biomedical Research Centre at the University Hospitals Bristol NHS Foundation Trust and the University of Bristol, Bristol, UK
5. K.G. Jebsen Center for Genetic Epidemiology, Department of Public Health and Nursing, NTNU, Norwegian University of Science and Technology, Norway.

Corresponding Author:

Alice Carter

[alice.carter@bristol.ac.uk](mailto:alice.carter@bristol.ac.uk)

Oakfield House,

Oakfield Grove,

Bristol,

BS8 2BN

0117 3310098

ORCiD ID: 0000-0003-2817-4195

[Mendelian randomisation for mediation analysis: current methods and challenges for implementation 1](#_Toc68774727)

[Supplementary methods 6](#_Toc68774728)

[Single mediators 6](#_Toc68774729)

[Multiple mediators 7](#_Toc68774730)

[Supplementary Tables 11](#_Toc68774731)

[sTable 1: Simulation scenarios 11](#_Toc68774732)

[sTable 2: Estimated effect sizes and size of bias for the effect of a continuous mediator between a continuous exposure and continuous outcome using the non-IV difference in coefficients (difference) method and product of coefficients (product) method (Simulated N=5000) 12](#_Toc68774733)

[sTable 3: Estimated effect sizes and size of bias for simulated effect of a continuous mediator explaining the effect between a continuous exposure and continuous outcome (per unit increase in exposure), and a rare binary outcome and common binary outcome using the non-IV difference in coefficients (difference) method and product of coefficients (product) method on the risk or mean difference scale with no residual covariance reflecting confounding (Simulated N=5000) 13](#_Toc68774734)

[sTable 4: Estimated effect sizes and size of bias for simulated effect of a continuous mediator explaining the effect between a continuous exposure and continuous outcome using Mendelian randomisation using the Mendelian randomisation methods multivariable Mendelian randomisation (MVMR) and two-step Mendelian randomisation (TSMR) (Simulated N=5000) 14](#_Toc68774735)

[sTable 5: Estimated effect sizes and size of bias for simulated effect of a continuous mediator explaining the effect between a continuous exposure and continuous outcome (per unit increase in exposure), and a rare binary outcome and common binary outcome on the risk or mean difference scale using the Mendelian randomisation methods multivariable Mendelian randomisation (MVMR) and two-step Mendelian randomisation (TSMR), where no residual covariance is included reflecting confounding (Simulated N=5000) 15](#_Toc68774736)

[sTable 6: Estimated effect sizes and size of bias for simulated effect of a continuous mediator explaining the effect between a continuous exposure and rare binary outcome using the non-IV difference in coefficients (difference) method and product of coefficients (product) method on the risk difference scale (Simulated N = 5000) 16](#_Toc68774737)

[sTable 7: Estimated effect sizes and size of bias for simulated effect of a continuous mediator explaining the effect between a continuous exposure and common binary outcome using the non-IV difference in coefficients (difference) method and product of coefficients (product) method on the risk difference scale (Simulated N=5000) 17](#_Toc68774738)

[sTable 8: Estimated effect sizes and size of bias for simulated effect of a continuous mediator explaining the effect between a continuous exposure and rare binary outcome using the non-IV difference in coefficients (difference) method and product of coefficients (product) method on the risk difference scale, where simulated total effects are small (Simulated N=5000) 18](#_Toc68774739)

[sTable 9: Estimated effect sizes and size of bias for simulated effect of a continuous mediator explaining the effect between a continuous exposure and common binary outcome using the non-IV difference in coefficients (difference) method and product of coefficients (product) method on the risk difference scale, where true total effects are small (Simulated N=5000) 19](#_Toc68774740)

[sTable 10: Estimated effect sizes and size of bias for simulated effect of a continuous mediator explaining the effect between a continuous exposure and a rare binary outcome using the Mendelian randomisation methods multivariable Mendelian randomisation (MVMR) and two-step Mendelian randomisation (TSMR) on the risk difference scale (Simulated N=5000) 20](#_Toc68774741)

[sTable 11: Estimated effect sizes and size of bias for simulated effect of a continuous mediator explaining the effect between a continuous exposure and a common binary outcome using the Mendelian randomisation methods multivariable Mendelian randomisation (MVMR) and two-step Mendelian randomisation (TSMR) on the risk difference scale (Simulated N=5000) 21](#_Toc68774742)

[sTable 12: Estimated effect sizes and size of bias for simulated effect of a continuous mediator explaining the effect between a continuous exposure and rare binary outcome using the Mendelian randomisation methods multivariable Mendelian randomisation (MVMR) and two-step Mendelian randomisation (TSMR) on the risk difference scale, where simulated total effects are small (Simulated N=5000) 22](#_Toc68774743)

[sTable 13: Estimated effect sizes and size of bias for simulated effect of a continuous mediator explaining the effect between a continuous exposure and common binary outcome using the Mendelian randomisation methods multivariable Mendelian randomisation (MVMR) and two-step Mendelian randomisation (TSMR) on the risk difference scale, where simulated total effects are small (Simulated N=5000) 23](#_Toc68774744)

[sTable 14: Estimated effect sizes and size of bias for simulated effect of a continuous mediator explaining the effect between a continuous exposure and a rare binary outcome using the Mendelian randomisation methods multivariable Mendelian randomisation (MVMR) and two-step Mendelian randomisation (TSMR) on the log odds ratio scale (Simulated N=5000) 24](#_Toc68774745)

[sTable 15: Estimated effect sizes and size of bias for simulated effect of a continuous mediator explaining the effect between a continuous exposure and a common binary outcome using the Mendelian randomisation methods multivariable Mendelian randomisation (MVMR) and two-step Mendelian randomisation (TSMR) on the log odds ratio scale (Simulated N=5000) 25](#_Toc68774746)

[sTable 16: Estimated effect sizes and size of bias for simulated effect of a continuous mediator explaining the effect between a continuous exposure and a rare binary outcome using the Mendelian randomisation methods multivariable Mendelian randomisation (MVMR) and two-step Mendelian randomisation (TSMR) on the odds ratio scale (Simulated N=5000) 26](#_Toc68774747)

[sTable 17: Estimated effect sizes and size of bias for simulated effect of a continuous mediator explaining the effect between a continuous exposure and a common binary outcome using the Mendelian randomisation methods multivariable Mendelian randomisation (MVMR) and two-step Mendelian randomisation (TSMR) on the odds ratio scale (Simulated N=5000) 27](#_Toc68774748)

[sTable 18: Estimated effect sizes and size of bias for simulated effect of a continuous mediator explaining the effect between a continuous exposure and continuous outcome (per unit increase in exposure), and a rare binary outcome and common binary outcome using the non-IV difference in coefficients (difference) method and product of coefficients (product) method on the risk or mean difference scale, where measurement error is introduced in either the exposure or mediator (Simulated N=5000) 28](#_Toc68774749)

[sTable 19: Estimated effect sizes and size of bias for simulated effect of a continuous mediator explaining the effect between a continuous exposure and continuous outcome (per unit increase in exposure), and a rare binary outcome and common binary outcome using the Mendelian randomisation methods multivariable Mendelian randomisation (MVMR) and two-step Mendelian randomisation (TSMR) on the risk or mean difference scale, where measurement error is introduced in either the exposure or mediator (Simulated N=5000) 29](#_Toc68774750)

[sTable 20: Estimated effect sizes and size of bias for simulated effect of a continuous mediator explaining the effect between a continuous exposure and continuous outcome (per unit increase in exposure), using the Mendelian randomisation methods multivariable Mendelian randomisation (MVMR) and two-step Mendelian randomisation (TSMR) on the mean difference scale, where pleiotropy is simulated in the exposure (Simulated N=5000) 30](#_Toc68774751)

[sTable 21: Estimated effect sizes and size of bias for simulated effect of a continuous mediator explaining the effect between a continuous exposure and continuous outcome (per unit increase in exposure), using the Mendelian randomisation methods multivariable Mendelian randomisation (MVMR) and two-step Mendelian randomisation (TSMR) on the mean difference scale, where pleiotropy is simulated in the mediator (Simulated N=5000) 31](#_Toc68774752)

[sTable 22: Estimated effect sizes and size of bias for simulated effect of a continuous mediator explaining the effect a continuous exposure and continuous outcome (per unit increase in exposure), and a rare binary outcome and common binary outcome using the Mendelian randomisation methods multivariable Mendelian randomisation (MVMR) and two-step Mendelian randomisation (TSMR) on the risk or mean difference scale, where simulated total effects are imprecise (Simulated N=1000) 32](#_Toc68774753)

[sTable 23: Estimated effect sizes and size of bias for simulated effect of a continuous mediator explaining the effect between a continuous exposure and continuous outcome using the Mendelian randomisation methods multivariable Mendelian randomisation (MVMR) and two-step Mendelian randomisation (TSMR) where true simulated total effects are small (Simulated N=5000) 33](#_Toc68774754)

[sTable 24: Estimated effect sizes and size of bias for simulated effect of a continuous mediator explaining the effect between a continuous exposure and continuous outcome, rare binary outcome and common binary outcome using the non-IV difference in coefficients (difference) method and product of coefficients (product) method on the risk or mean difference scale, where simulated total effects are imprecise (Simulated N=1000) 34](#_Toc68774755)

[sTable 25: Estimated effect sizes and size of bias for simulated effect of a continuous mediator explaining the effect between a continuous exposure and continuous outcome using the non-IV difference in coefficients (difference) method and product of coefficients (product) method on the mean difference scale where true total effects simulated are small (Simulated N=5000) 35](#_Toc68774756)

[sTable 26: Estimated total effect and direct effect of the exposure on a continuous outcome mediated by a continuous mediator, where an interaction between the exposure and mediator is present using the non-IV difference in coefficients (difference) method and multivariable Mendelian randomisation (MVMR) method (Simulated N = 5000) 36](#_Toc68774757)

[sTable 27: Estimated indirect effect and proportion mediated by multiple continuous mediators explaining the association between a continuous exposure and continuous outcome in simulation analyses using non-IV methods and Mendelian randomisation methods (Simulated N = 5000) 37](#_Toc68774758)

[Supplementary Figures 38](#_Toc68774759)

[sFigure 1: Directed acyclic graph illustrating Mendelian randomisation and the instrumental variable assumptions required for valid inference 38](#_Toc68774760)

[sFigure 2: Directed acyclic graphs depicting simulation scenarios considering the role of multiple mediators where in A) all three mediators are independent and in B) there is covariance between two of the three mediators 39](#_Toc68774761)

[sFigure 3: Directed acyclic graphs depicting how collider bias can be introduced in non-IV mediation analysis when conditioning on a mediator in the presence of un- or mis- measured mediator-outcome confounders 40](#_Toc68774762)

[sFigure 4: Estimates of the proportion mediated and size of absolute bias when weak instrument bias is simulated in A) the exposure and B) the mediator for a true proportion mediated of 0.25 (solid line) (simulated N = 5000) 41](#_Toc68774763)

[References 42](#_Toc68774764)

# Supplementary methods

Using the notation X = exposure, M = mediator, M1 = mediator 1, M2 = mediator 2, M3 – mediator 3, Y = outcome, G = genetic instruments, C = measured confounders, V = uncorrelated error term, µ = uncorrelated error term, four methods are compared (figure 1). Here we give the regression equations to estimate each of the methods described in the main paper. Notation and equations for the difference method and product of coefficients method are adapted from Vanderweele, 2015, where full details of the equations and notations are available [1]. Variables and parameters given in bold indicate the main coefficient(s) of interest in each case.

### Single mediators

1. The difference method to estimate the direct effect (and infer the indirect effect) using non-instrumental variable (IV) observed data

**Total:**

Y = θ^+^_0_ + **θ^+^_1_X** + θ^+^_3_C

**Direct:**

Y = θ_0_ + **θ_1_X** + θ_2_M + θ_4_C

**Indirect:**

**θ^+^_1_ - θ_1_**

1. The product of coefficients method to estimate the indirect effect using non-IV observed data

**Exposure-Mediator:**

M = β_0_ + **β_1_X** + β_3_C

**Direct:**

Y= θ_0_ + **θ_1_X** + θ_2_M + θ_4_C

**Indirect:**

**β_1_θ_2_**

1. Multivariable MR to estimate the direct effect and indirect effect using a single genetic instrumental variable for each of the exposure and mediator, using two-stage least squares regression

**Total:**

X = π_0_ + π_1_G_x_ + v_1_

Y = β_0_ + **β_XT_X** + µ_1_

**Direct:**

X = π_0_ + π_1x_G_x_ + π_2x_G_M_ + v_1_

M = π_0_ + π_1z_G_x_ + π_2z_G_M_ + v_2_

Y = β_0_ + **β_X_X** + β_M_M + µ_2_

**Indirect:**

**β_XT_- β_X_**

1. Two-step MR to estimate the indirect effect using genetic instrumental variables for both the exposure and mediator, using two-stage least squares regression

**Exposure-Mediator:**

X = π_0_ + π_1_G_x_ + v_1_

M = β_0_ + **β_XM_X** + µ_1_

**Direct**:

X = π_0_ + π_1x_G_x_ + π_2x_G_M_ + v_1_

M = π_0_ + π_1z_G_x_ + π_2z_G_M_ + v_2_

Y = β_0_ + **β_X_X** + β_M_M + µ_2_

**Indirect**:

**β_XM_ β_M_**

### Multiple mediators

1. The difference method to estimate the direct effect and indirect effect using non-IV observed data mutually adjusting for all mediators

**Total:**

Y = θ^+^_0_ + **θ^+^_1_X** + θ^+^_5_C

**Direct:**

Y = θ_0_ + **θ_1_X** + θ_2_M1 + θ_3_M2 + θ_4_M3 + θ_5_C

**Indirect:**

**θ^+^_1_X - θ_1_X**

1. The product of coefficients method to estimate the indirect effect using non-IV observed data, considering each mediator individually

**Mediator 1:**

**Exposure-Mediator:**

M1= β_0_ + β_1_X + β_4_C

**Direct:**

Y= θ_0_ + **θ_1_X** + θ_2M1_M1 + θ_4_C

**Indirect:**

**β_1_θ_2M1_**

**Mediator 2:**

**Exposure-Mediator:**

M2= β_0_ + **β_2_X** + β_4_C

**Direct:**

Y= θ_0_ + **θ_1_X** + θ_2M2_M2 + θ_4_C

**Indirect:**

**β_2_θ_2M2_**

**Mediator 3:**

**Exposure-Mediator:**

M3= β_0_ + β_3_X + β_4_C

**Direct:**

Y= θ_0_ + **θ_1_X** + θ_2M3_M3 + θ_4_C

**Indirect:**

**β_3_θ_2M3_**

**Combined indirect:**

**β_1_θ_2M1_ + β_2_θ_2M2_ + β_3_θ_2M3_**

1. Multivariable MR to estimate the direct effect and indirect effect using a single genetic instrumental variable for each of the exposure and mediator, using two-stage least squares regression

**Total:**

X = π_0_ + π_1_G_x_ + v_1_

Y = β_0_ + **β_XT_X** + µ_1_

**Direct:**

X = π_0_ + π_1x_G_x_ + π_2x_G_M1_+ π_3x_G_M2_+ π_4x_G_M3_ + v_1_

M1 = π_1_ + π_1z_G_x_ + π_2z_G_M1_+ π_3z_G_M2_+ π_4z_G_M3_ + v_2_

M2 = π_2_ + π_1Ω_G_x_ + π_2Ω_G_M1_+ π_3Ω_G_M2_+ π_4Ω_G_M3_ + v_3_

M3 = π_3_ + π_3α_G_x_ + π_2α_G_M1_+ π_2α_G_M2_+ π_4α_G_M3_ + v_4_

Y = β_0_ + **β_X_X** + β_M1_M1 + β_M2_M2 + β_M3_M3 + µ_2_

**Indirect:**

**β_XT_ - β_X_**

1. Two-step MR to estimate the indirect effect using genetic instrumental variables for both the exposure and mediator, using two-stage least squares regression

**Mediator 1:**

**Exposure-Mediator:**

X = π_0_ + π_1_G_x_ + v_X_

M1 = β_0_ + **β_XM1_X** + µ_1_

**Direct:**

X = π_0_ + π_1x_G_x_ + π_2x_G_M1_ + v_X1_

M1= π_01_ + π_11_G_x_ + π_21_G_M1_ + v_M1_

Y = β_0_ + **β_X1_X** + β_M1_M1 + µ_2_

**Indirect:**

**β_XM1_β_M1_**

**Mediator 2:**

**Exposure-Mediator:**

X = π_0_ + π_1_G_x_ + v_X_

M2 = β_0_ + **β_xM2_X** + µ_3_

**Direct:**

X = π_02_ + π_12_G_x_ + π_22_G_M2_ + v_X2_

M2 = π_0M2_ + π_1M2_G_x_ + π_2M2_G_M2_ + v_M2_

Y = β_1_ + **β_X2_X** + β_M2_M2 + µ_4_

**Indirect:**

**β_XM2_β_M2_**

**Mediator 3:**

**Exposure-Mediator:**

X = π_0_ + π_1_G_x_ + v_X_

M3 = β_0_ + **β_XM3_X** + µ_3_

**Direct:**

X = π_03_ + π_13_**G**_x_ + π_23_G_M3_ + v_X3_

M3 = π_0M3_ + π_1M3_G_x_ + π_2M3_**G_M2_** + v_M3_

Y = β_2_ + **β_X3_X** + β_M3_M3 + µ_6_

**Indirect:**

**β_XM3_β_M3_**

**Combined indirect:**

**β_XM1_β_M1_ + β_XM2_β_M2_+ β_XM3_β_M3_**

# Supplementary Tables

## sTable 1: Simulation scenarios

|  | **Total effect** | **Proportion mediated** | | | | | **Sample Size** | **Measurement error** | **Weak instrument** | **Pleiotropy** |
| --- | --- | --- | --- | --- | --- | --- | --- | --- | --- | --- |
| No Mediation | 0.5 | 0 |  |  |  |  | 5000 |  |  |  |
| Inconsistent mediation | 0.5 | -0.5 |  |  |  |  |  |  |  |  |
|  | | | | | | | | | |  |
| Varying total effect | 0 | 0.05 | 0.25 | 0.75 |  |  | 5000 |  |  |  |
|  | 0.2 | 0.05 | 0.25 | 0.75 |  |  |  |  |  |  |
|  | 0.5 | 0.05 | 0.25 | 0.75 |  |  |  |  |  |  |
|  | 1 | 0.05 | 0.25 | 0.75 |  |  |  |  |  |  |
|  | | | | | | | | | |  |
| Small total effect | 0.01 | 0.05 | 0.25 | 0.75 |  |  | 5000 |  |  |  |
|  | 0.05 | 0.05 | 0.25 | 0.75 |  |  |  |  |  |  |
|  | 0.1 | 0.05 | 0.25 | 0.75 |  |  |  |  |  |  |
|  | | | | | | | | | |  |
| Imprecise total effect | 0.2 | 0.05 | 0.25 | 0.75 |  |  | 1000 |  |  |  |
|  | | | | | | | | | |  |
| Measurement error | 0.5 | 0.25 |  |  |  |  | 5000 | Exposure |  |  |
|  | 0.5 | 0.25 |  |  |  |  |  | Mediator |  |  |
|  | | | | | | | | | |  |
| Weak instrument bias | 0.5 | 0.25 |  |  |  |  | 5000 |  | Exposure |  |
|  | 0.5 | 0.25 |  |  |  |  |  |  | Mediator |  |
|  | | | | | | | | | |  |
| Pleiotropy | 0.2 | 0.05 | 0.25 | 0.75 |  |  | 5000 |  |  | Association between exposure and outcome |
|  | 0.5 | 0.05 | 0.25 | 0.75 |  |  |  |  |  |  |
|  | 1.0 | 0.05 | 0.25 | 0.75 |  |  |  |  |  |  |
|  | 0.2 | 0.05 | 0.25 | 0.75 |  |  |  |  |  | Association between mediator and outcome |
|  | 0.5 | 0.05 | 0.25 | 0.75 |  |  |  |  |  |  |
|  | 1.0 | 0.05 | 0.25 | 0.75 |  |  |  |  |  |  |
|  | | | | | | | | | | |
| No confounding* | 0 | 0.05 | 0.25 | 0.75 |  |  | 5000 |  |  |  |
|  | | | | | | | | | |  |
| Multiple mediators |  | **Joint** | **M1** | **M2** | **M3** | **M3 via M2** | 5000 |  |  |  |
|  | 0.45 | 0.56 | 0.11 | 0.18 | 0.12 | 0 |  |  |  |  |
|  |  | 0.56 | 0.11 | 0.18 | 0.27 | 0.06 |  |  |  |  |
|  | | | | | | | | | | |
| X*M Interactions | **Direct effect** | **Interaction effect** | | | | | 5000 |  |  |  |
|  | 0.4 | 0.05 | | 0.01 | | 0.2 |  |  |  |  |

In all simulations the effect of the mediator on the outcome is set to 0.2. All simulations undergo 1000 replications.

Confounding is simulated as residual covariance between the exposure, mediator and outcome in all scenarios except*

## sTable 2: Estimated effect sizes and size of bias for the effect of a continuous mediator between a continuous exposure and continuous outcome using the non-IV difference in coefficients (difference) method and product of coefficients (product) method (Simulated N=5000)

| **Mediation method** | **True proportion mediated** | **True total effect** | **Total effect (SD)** | **Size of bias (absolute)** | **Size of bias (relative)** | **Direct effect (SD)** | **Size of bias (absolute)** | **Size of bias (relative)** | **Indirect effect (SD)** | **Size of bias (absolute)** | **Size of bias (relative)** | **Proportion mediated (SD)** | **Size of bias (absolute)** | **Size of bias (relative)** |
| --- | --- | --- | --- | --- | --- | --- | --- | --- | --- | --- | --- | --- | --- | --- |
| Difference | 0 | 0.5 | 1.1 (0.009) | 0.60 | 1.20 | 0.833 (0.007) | 0.33 | 0.67 | 0.267 (0.007) | 0.27 | NA | 0.243 (0.006) | 0.24 | NA |
| Product |  |  |  |  |  |  |  |  | 0.267 (0.007) | 0.27 | NA | 0.243 (0.006) | 0.24 | 0.12 |
| Difference | -0.5 | 0.5 | 1.1 (0.009) | 0.60 | 1.20 | 1.5 (0.007) | 0.75 | 1.50 | -0.4 (0.008) | -1.15 | 4.60 | -0.364 (0.009) | 0.14 | -0.27 |
| Product |  |  |  |  |  |  |  |  | -0.4 (0.008) | -1.15 | 4.60 | -0.364 (0.009) | 0.14 | 0.07 |
| Difference | 0.05 | 0 | 0.6 (0.009) | 0.60 | NA | 0.333 (0.007) | 0.33 | NA | 0.267 (0.007) | 0.27 | NA | 0.445 (0.009) | 0.39 | 7.90 |
| Product |  |  |  |  |  |  |  |  | 0.267 (0.007) | 0.27 | NA | 0.445 (0.009) | 0.39 | 0.20 |
| Difference |  | 0.2 | 0.8 (0.009) | 0.80 | 4.00 | 0.507 (0.007) | 0.32 | 1.58 | 0.293 (0.007) | 0.28 | 28.33 | 0.367 (0.007) | 0.32 | 6.33 |
| Product |  |  |  |  |  |  |  |  | 0.293 (0.007) | 0.28 | 28.33 | 0.367 (0.007) | 0.32 | 0.16 |
| Difference |  | 0.5 | 1.1 (0.009) | 0.90 | 1.80 | 0.767 (0.007) | 0.29 | 0.58 | 0.333 (0.008) | 0.31 | 12.32 | 0.303 (0.006) | 0.25 | 5.05 |
| Product |  |  |  |  |  |  |  |  | 0.333 (0.008) | 0.31 | 12.32 | 0.303 (0.006) | 0.25 | 0.13 |
| Difference |  | 1 | 1.6 (0.009) | 1.10 | 1.10 | 1.2 (0.007) | 0.25 | 0.25 | 0.4 (0.008) | 0.35 | 7.00 | 0.25 (0.004) | 0.20 | 4.00 |
| Product |  |  |  |  |  |  |  |  | 0.4 (0.008) | 0.35 | 7.00 | 0.25 (0.004) | 0.20 | 0.10 |
| Difference | 0.25 | 0 | 0.6 (0.008) | 0.60 | NA | 0.334 (0.006) | 0.33 | NA | 0.267 (0.007) | 0.27 | NA | 0.444 (0.009) | 0.19 | 0.78 |
| Product |  |  |  |  |  |  |  |  | 0.267 (0.007) | 0.27 | NA | 0.444 (0.009) | 0.19 | 0.10 |
| Difference |  | 0.2 | 0.8 (0.009) | 0.80 | 4.00 | 0.4 (0.007) | 0.25 | 1.25 | 0.399 (0.008) | 0.35 | 6.99 | 0.499 (0.008) | 0.25 | 1.00 |
| Product |  |  |  |  |  |  |  |  | 0.399 (0.008) | 0.35 | 6.99 | 0.499 (0.008) | 0.25 | 0.12 |
| Difference |  | 0.5 | 1.1 (0.009) | 0.90 | 1.80 | 0.5 (0.01) | 0.12 | 0.25 | 0.6 (0.01) | 0.48 | 3.80 | 0.546 (0.008) | 0.30 | 1.18 |
| Product |  |  |  |  |  |  |  |  | 0.6 (0.01) | 0.48 | 3.80 | 0.546 (0.008) | 0.30 | 0.15 |
| Difference |  | 1 | 1.6 (0.009) | 1.10 | 1.10 | 0.667 (0.013) | -0.08 | -0.08 | 0.933 (0.014) | 0.68 | 2.73 | 0.583 (0.008) | 0.33 | 1.33 |
| Product |  |  |  |  |  |  |  |  | 0.933 (0.014) | 0.68 | 2.73 | 0.583 (0.008) | 0.33 | 0.17 |
| Difference | 0.75 | 0 | 0.6 (0.009) | 0.60 | NA | 0.333 (0.007) | 0.33 | NA | 0.267 (0.007) | 0.27 | NA | 0.445 (0.009) | -0.31 | -0.41 |
| Product |  |  |  |  |  |  |  |  | 0.267 (0.007) | 0.27 | NA | 0.445 (0.009) | -0.31 | -0.15 |
| Difference |  | 0.2 | 0.8 (0.009) | 0.80 | 4.00 | 0.134 (0.01) | 0.08 | 0.42 | 0.666 (0.011) | 0.52 | 3.44 | 0.833 (0.012) | 0.08 | 0.11 |
| Product |  |  |  |  |  |  |  |  | 0.666 (0.011) | 0.52 | 3.44 | 0.833 (0.012) | 0.08 | 0.04 |
| Difference |  | 0.5 | 1.1 (0.008) | 0.90 | 1.80 | -0.166 (0.017) | -0.29 | -0.58 | 1.267 (0.017) | 0.89 | 2.38 | 1.151 (0.016) | 0.40 | 0.54 |
| Product |  |  |  |  |  |  |  |  | 1.267 (0.017) | 0.89 | 2.38 | 1.151 (0.016) | 0.40 | 0.20 |
| Difference |  | 1 | 1.6 (0.009) | 1.10 | 1.10 | -0.666 (0.03) | -0.92 | -0.92 | 2.266 (0.03) | 1.52 | 2.02 | 1.416 (0.019) | 0.67 | 0.89 |
| Product |  |  |  |  |  |  |  |  | 2.266 (0.03) | 1.52 | 2.02 | 1.416 (0.019) | 0.67 | 0.33 |

## sTable 3: Estimated effect sizes and size of bias for simulated effect of a continuous mediator explaining the effect between a continuous exposure and continuous outcome (per unit increase in exposure), and a rare binary outcome and common binary outcome using the non-IV difference in coefficients (difference) method and product of coefficients (product) method on the risk or mean difference scale with no residual covariance reflecting confounding (Simulated N=5000)

| **Outcome** | **Mediation method** | **True total effect** | **True proportion mediated** | **Total effect (SD)** | **Size of bias (absolute)** | **Direct effect (SD)** | **Size of bias (absolute)** | **Indirect effect(SD)** | **Size of bias (absolute)** | **Proportion mediated**  **(SD)** | **Size of bias (absolute)** | **Size of bias (relative)** |
| --- | --- | --- | --- | --- | --- | --- | --- | --- | --- | --- | --- | --- |
| **Continuous outcome** | Difference | 0 | 0.05 | 0 (0.01) | 0.00 | 0 (0.01) | 0.00 | 0 (0.01) | 0.00 | 0 (0.01) | 0.18 | 3.53 |
|  | Product |  |  |  |  |  |  | 0 (0.003) | 0.00 | 0.227 (10.342) | 0.18 | 0.09 |
|  | Difference |  | 0.25 | 0 (0.01) | 0.00 | 0 (0.01) | 0.00 | 0 (0.01) | 0.00 | 0 (0.01) | -0.10 | -0.39 |
|  | Product |  |  |  |  |  |  | 0 (0.003) | 0.00 | 0.152 (3.448) | -0.10 | -0.05 |
|  | Difference |  | 0.75 | 0 (0.01) | 0.00 | 0 (0.01) | 0.00 | 0 (0.01) | 0.00 | 0 (0.01) | -1.18 | -1.57 |
|  | Product |  |  |  |  |  |  | 0 (0.003) | 0.00 | -0.427 (24.509) | -1.18 | -0.59 |
| **Rare binary outcome** | Difference | 0 | 0.05 | 0 (0.002) | 0.00 | 0 (0.002) | 0.00 | 0 (0.002) | 0.00 | 0 (0.002) | -0.39 | -7.83 |
|  | Product |  |  |  |  |  |  | 0 (0) | 0.00 | -0.342 (13.323) | -0.39 | -7.83 |
|  | Difference |  | 0.25 | 0 (0.002) | 0.00 | 0 (0.002) | 0.00 | 0 (0.002) | 0.00 | 0 (0.002) | -0.18 | -0.71 |
|  | Product |  |  |  |  |  |  | 0 (0) | 0.00 | 0.073 (3.212) | -0.18 | -0.71 |
|  | Difference |  | 0.75 | 0 (0.002) | 0.00 | 0 (0.002) | 0.00 | 0 (0.002) | 0.00 | 0 (0.002) | -0.48 | -0.65 |
|  | Product |  |  |  |  |  |  | 0 (0) | 0.00 | 0.266 (5.102) | -0.48 | -0.65 |
| **Common binary outcome** | Difference | 0 | 0.05 | 0 (0.004) | 0.00 | 0 (0.004) | 0.00 | 0 (0.004) | 0.00 | 0 (0.004) | -0.01 | -0.11 |
|  | Product |  |  |  |  |  |  | 0 (0.001) | 0.00 | 0.044 (3.642) | -0.01 | -0.11 |
|  | Difference |  | 0.25 | 0 (0.004) | 0.00 | 0 (0.004) | 0.00 | 0 (0.004) | 0.00 | 0 (0.004) | -0.13 | -0.50 |
|  | Product |  |  |  |  |  |  | 0 (0.001) | 0.00 | 0.125 (4.933) | -0.13 | -0.50 |
|  | Difference |  | 0.75 | 0 (0.004) | 0.00 | 0 (0.004) | 0.00 | 0 (0.004) | 0.00 | 0 (0.004) | -0.64 | -0.85 |
|  | Product |  |  |  |  |  |  | 0 (0.001) | 0.00 | 0.114 (9.155) | -0.64 | -0.85 |

Note: Relative bias cannot be estimated for the total effect direct effect and indirect effect because there is no true total effect

## sTable 4: Estimated effect sizes and size of bias for simulated effect of a continuous mediator explaining the effect between a continuous exposure and continuous outcome using Mendelian randomisation using the Mendelian randomisation methods multivariable Mendelian randomisation (MVMR) and two-step Mendelian randomisation (TSMR) (Simulated N=5000)

| **Mediation method** | **True prop-ortion mediated** | **True total effect** | **Total effect (SD)** | **Size of bias (absolute)** | **Size of bias (relative)** | **Direct effect (SD)** | **Size of bias (absolute)** | **Size of bias (relative)** | **Indirect effect (SD)** | **Size of bias (absolute)** | **Size of bias (relative)** | **Proportion mediated (SD)** | **Size of bias (absolute)** | **Size of bias (relative)** |
| --- | --- | --- | --- | --- | --- | --- | --- | --- | --- | --- | --- | --- | --- | --- |
| MVMR | 0 | 0.5 | 0.499 (0.017) | 0.00 | 0.00 | 0.5 (0.014) | 0.00 | 0.00 | 0 (0.004) | 0.00 | NA | 0 (0.008) | 0.00 | NA |
| TSMR |  |  |  |  |  |  |  |  | 0 (0.004) | 0.00 | NA | 0.004 (0) | 0.00 | NA |
| MVMR | -0.5 | 0.5 | 0.499 (0.017) | 0.00 | 0.00 | 0.75 (0.023) | 0.00 | 0.00 | -0.25 (0.018) | 0.00 | 0.00 | -0.501 (0.043) | 0.00 | 0.00 |
| TSMR |  |  |  |  |  |  |  |  | -0.25 (0.018) | 0.00 | 0.00 | -0.501 (0.043) | 0.00 | 0.00 |
| MVMR | 0.05 | 0 | 0 (0.017) | 0.00 | NA | 0 (0.014) | 0.00 | NA | 0 (0.004) | 0.00 | NA | 0.164 (2.176) | 0.11 | 2.28 |
| TSMR |  |  |  |  |  |  |  |  | 0 (0.004) | 0.00 | NA | 0.004 (0.164) | 0.11 | 2.28 |
| MVMR |  | 0.2 | 0.2 (0.017) | 0.00 | 0.00 | 0.19 (0.014) | 0.00 | 0.00 | 0.01 (0.004) | 0.00 | 0.01 | 0.049 (0.017) | 0.00 | -0.01 |
| TSMR |  |  |  |  |  |  |  |  | 0.01 (0.004) | 0.00 | 0.01 | 0.004 (0.049) | 0.00 | -0.01 |
| MVMR |  | 0.5 | 0.5 (0.018) | 0.00 | 0.00 | 0.475 (0.015) | 0.00 | 0.00 | 0.025 (0.004) | 0.00 | -0.01 | 0.05 (0.008) | 0.00 | -0.01 |
| TSMR |  |  |  |  |  |  |  |  | 0.025 (0.004) | 0.00 | -0.01 | 0.004 (0.05) | 0.00 | -0.01 |
| MVMR |  | 1 | 1 (0.017) | 0.00 | 0.00 | 0.95 (0.014) | 0.00 | 0.00 | 0.05 (0.005) | 0.00 | 0.00 | 0.05 (0.005) | 0.00 | 0.00 |
| TSMR |  |  |  |  |  |  |  |  | 0.05 (0.005) | 0.00 | 0.00 | 0.005 (0.05) | 0.00 | 0.00 |
| MVMR | 0.25 | 0 | 0 (0.018) | 0.00 | NA | 0 (0.014) | 0.00 | NA | 0 (0.004) | 0.00 | NA | 0.111 (3.554) | -0.14 | -0.56 |
| TSMR |  |  |  |  |  |  |  |  | 0 (0.004) | 0.00 | NA | 0.004 (0.111) | -0.14 | -0.56 |
| MVMR |  | 0.2 | 0.199 (0.017) | 0.00 | -0.01 | 0.149 (0.014) | 0.00 | 0.00 | 0.049 (0.005) | 0.00 | -0.01 | 0.249 (0.022) | 0.00 | 0.00 |
| TSMR |  |  |  |  |  |  |  |  | 0.049 (0.005) | 0.00 | -0.01 | 0.005 (0.249) | 0.00 | 0.00 |
| MVMR |  | 0.5 | 0.5 (0.017) | 0.00 | 0.00 | 0.375 (0.017) | 0.00 | 0.00 | 0.125 (0.01) | 0.00 | 0.00 | 0.25 (0.019) | 0.00 | 0.00 |
| TSMR |  |  |  |  |  |  |  |  | 0.125 (0.01) | 0.00 | 0.00 | 0.01 (0.25) | 0.00 | 0.00 |
| MVMR |  | 1 | 1.001 (0.017) | 0.00 | 0.00 | 0.751 (0.023) | 0.00 | 0.00 | 0.249 (0.018) | 0.00 | 0.00 | 0.249 (0.018) | 0.00 | 0.00 |
| TSMR |  |  |  |  |  |  |  |  | 0.249 (0.018) | 0.00 | 0.00 | 0.018 (0.249) | 0.00 | 0.00 |
| MVMR | 0.75 | 0 | 0 (0.017) | 0.00 | NA | 0 (0.014) | 0.00 | NA | 0 (0.004) | 0.00 | NA | -0.046 (4.949) | -0.80 | -1.06 |
| TSMR |  |  |  |  |  |  |  |  | 0 (0.004) | 0.00 | NA | 0.004 (-0.046) | -0.80 | -1.06 |
| MVMR |  | 0.2 | 0.2 (0.018) | 0.00 | 0.00 | 0.05 (0.018) | 0.00 | 0.00 | 0.15 (0.011) | 0.00 | 0.00 | 0.754 (0.074) | 0.00 | 0.01 |
| TSMR |  |  |  |  |  |  |  |  | 0.15 (0.011) | 0.00 | 0.00 | 0.011 (0.754) | 0.00 | 0.01 |
| MVMR |  | 0.5 | 0.501 (0.017) | 0.00 | 0.00 | 0.126 (0.029) | 0.00 | 0.01 | 0.374 (0.026) | 0.00 | 0.00 | 0.748 (0.056) | 0.00 | 0.00 |
| TSMR |  |  |  |  |  |  |  |  | 0.374 (0.026) | 0.00 | 0.00 | 0.026 (0.748) | 0.00 | 0.00 |
| MVMR |  | 1 | 1 (0.017) | 0.00 | 0.00 | 0.249 (0.057) | 0.00 | 0.00 | 0.751 (0.055) | 0.00 | 0.00 | 0.751 (0.056) | 0.00 | 0.00 |
| TSMR |  |  |  |  |  |  |  |  | 0.751 (0.055) | 0.00 | 0.00 | 0.055 (0.751) | 0.00 | 0.00 |

Total effect = estimated using univariate Mendelian randomisation; direct effect = estimated using multivariable Mendelian randomisation controlling for both exposure and mediator

## sTable 5: Estimated effect sizes and size of bias for simulated effect of a continuous mediator explaining the effect between a continuous exposure and continuous outcome (per unit increase in exposure), and a rare binary outcome and common binary outcome on the risk or mean difference scale using the Mendelian randomisation methods multivariable Mendelian randomisation (MVMR) and two-step Mendelian randomisation (TSMR), where no residual covariance is included reflecting confounding (Simulated N=5000)

|  | **Mediation method** | **True total effect** | **True proportion mediated** | **Total effect (SD)** | **Size of bias (absolute)** | **Direct effect (SD)** | **Size of bias (absolute)** | **Indirect effect (SD)** | **Size of bias (absolute)** | **Proportion mediated (SD)** | **Size of bias (absolute)** | **Size of bias (relative)** |
| --- | --- | --- | --- | --- | --- | --- | --- | --- | --- | --- | --- | --- |
| Continuous outcome | MVMR | 0 | 0.05 | 0 (0.003) | 0.00 | 0.227 (10.342) | 0.00 | 0 (0.015) | 0.00 | 0.811 (24.209) | 0.76 | 15.22 |
|  | TSMR |  |  |  |  |  |  | 0 (0.004) | 0.00 | 0.811 (24.209) | 0.76 | 15.22 |
|  | MVMR |  | 0.25 | 0 (0.003) | 0.00 | 0.152 (3.448) | 0.00 | 0 (0.015) | 0.00 | -0.672 (31.009) | -0.92 | -3.69 |
|  | TSMR |  |  |  |  |  |  | 0 (0.004) | 0.00 | -0.672 (31.009) | -0.92 | -3.69 |
|  | MVMR |  | 0.75 | 0 (0.003) | 0.00 | -0.427 (24.509) | 0.00 | 0 (0.015) | 0.00 | -0.022 (5.39) | -0.77 | -1.03 |
|  | TSMR |  |  |  |  |  |  | 0 (0.004) | 0.00 | -0.022 (5.39) | -0.77 | -1.03 |
| Rare binary outcome | MVMR | 0 | 0.05 | 0 (0) | 0.00 | -0.342 (13.323) | 0.00 | 0 (0.003) | 0.00 | 0 (0.003) | 1.29 | 25.90 |
|  | TSMR |  |  |  |  |  |  | 0 (0) | 0.00 | 1.345 (43.052) | 1.29 | 25.90 |
|  | MVMR |  | 0.25 | 0 (0) | 0.00 | 0.073 (3.212) | 0.00 | 0 (0.003) | 0.00 | 0 (0.003) | -0.21 | -0.85 |
|  | TSMR |  |  |  |  |  |  | 0 (0) | 0.00 | 0.039 (1.697) | -0.21 | -0.85 |
|  | MVMR |  | 0.75 | 0 (0) | 0.00 | 0.266 (5.102) | 0.00 | 0 (0.003) | 0.00 | 0 (0.003) | -0.81 | -1.08 |
|  | TSMR |  |  |  |  |  |  | 0 (0) | 0.00 | -0.059 (3.035) | -0.81 | -1.08 |
| Common binary outcome | MVMR | 0 | 0.05 | 0 (0.001) | 0.00 | 0.044 (3.642) | 0.00 | 0 (0.006) | 0.00 | 0 (0.006) | 0.78 | 15.57 |
|  | TSMR |  |  |  |  |  |  | 0 (0.001) | 0.00 | 0.829 (30.515) | 0.78 | 15.57 |
|  | MVMR |  | 0.25 | 0 (0.001) | 0.00 | 0.125 (4.933) | 0.00 | 0 (0.006) | 0.00 | 0 (0.006) | -0.24 | -0.96 |
|  | TSMR |  |  |  |  |  |  | 0 (0.001) | 0.00 | 0.01 (4.135) | -0.24 | -0.96 |
|  | MVMR |  | 0.75 | 0 (0.001) | 0.00 | 0.114 (9.155) | 0.00 | 0 (0.006) | 0.00 | 0 (0.006) | -0.19 | -0.25 |
|  | TSMR |  |  |  |  |  |  | 0 (0.001) | 0.00 | 0.565 (59.327) | -0.19 | -0.25 |

Total effect = estimated using univariate Mendelian randomisation; direct effect = estimated using multivariable Mendelian randomisation controlling for both exposure and mediator

## sTable 6: Estimated effect sizes and size of bias for simulated effect of a continuous mediator explaining the effect between a continuous exposure and rare binary outcome using the non-IV difference in coefficients (difference) method and product of coefficients (product) method on the risk difference scale (Simulated N = 5000)

| **Mediation method** | **True proportion mediated** | **True total effect** | **Total effect (SD)** | **Size of bias (absolute)** | **Size of bias (relative)** | **Direct effect (SD)** | **Size of bias (absolute)** | **Size of bias (relative)** | **Indirect effect (SD)** | **Size of bias (absolute)** | **Size of bias (relative)** | **Proportion mediated (SD)** | **Size of bias (absolute)** | **Size of bias (relative)** |
| --- | --- | --- | --- | --- | --- | --- | --- | --- | --- | --- | --- | --- | --- | --- |
| Difference | 0 | 0.025 | 0.064 (0.001) | 0.04 | 1.54 | 0.048 (0.002) | 0.02 | 0.92 | 0.015 (0.001) | 0.02 | NA | 0.244 (0.019) | 0.24 | NA |
| Product |  |  |  |  |  |  |  |  | 0.015 (0.001) | 0.02 | NA | 0.244 (0.019) | 0.24 | NA |
| Difference | -0.5 |  | 0.064 (0.001) | 0.04 | 1.54 | 0.087 (0.002) | 0.05 | 1.31 | -0.023 (0.002) | -0.06 | 4.86 | -0.365 (0.029) | 0.13 | -0.27 |
| Product |  |  |  |  |  |  |  |  | -0.023 (0.002) | -0.06 | 4.86 | -0.365 (0.029) | 0.13 | -0.27 |
| Difference | 0.05 | 0 | 0.051 (0.002) | 0.05 | NA | 0.028 (0.002) | 0.03 | NA | 0.023 (0.001) | 0.02 | NA | 0.447 (0.029) | 0.40 | 7.93 |
| Product |  |  |  |  |  |  |  |  | 0.023 (0.001) | 0.02 | NA | 0.447 (0.029) | 0.40 | 7.93 |
| Difference |  | 0.1 | 0.058 (0.001) | -0.04 | -0.42 | 0.037 (0.002) | -0.06 | -0.61 | 0.021 (0.001) | 0.02 | 3.24 | 0.367 (0.025) | 0.32 | 6.35 |
| Product |  |  |  |  |  |  |  |  | 0.021 (0.001) | 0.02 | 3.24 | 0.367 (0.025) | 0.32 | 6.35 |
| Difference |  | 0.025 | 0.064 (0.001) | 0.04 | 1.54 | 0.044 (0.002) | 0.02 | 0.86 | 0.019 (0.001) | 0.02 | 14.43 | 0.304 (0.025) | 0.25 | 5.07 |
| Product |  |  |  |  |  |  |  |  | 0.019 (0.001) | 0.02 | 14.43 | 0.304 (0.025) | 0.25 | 5.07 |
| Difference |  | 0.05 | 0.068 (0.001) | 0.02 | 0.36 | 0.051 (0.002) | 0.00 | 0.07 | 0.017 (0.002) | 0.01 | 5.83 | 0.251 (0.026) | 0.20 | 4.02 |
| Product |  |  |  |  |  |  |  |  | 0.017 (0.002) | 0.01 | 5.83 | 0.251 (0.026) | 0.20 | 4.02 |
| Difference | 0.25 | 0 | 0.051 (0.002) | 0.05 | NA | 0.028 (0.002) | 0.03 | NA | 0.023 (0.001) | 0.02 | NA | 0.445 (0.028) | 0.19 | 0.78 |
| Product |  |  |  |  |  |  |  |  | 0.023 (0.001) | 0.02 | NA | 0.445 (0.028) | 0.19 | 0.78 |
| Difference |  | 0.1 | 0.058 (0.001) | -0.04 | -0.21 | 0.029 (0.002) | -0.05 | -0.61 | 0.029 (0.002) | 0.00 | 0.16 | 0.5 (0.034) | 0.25 | 1.00 |
| Product |  |  |  |  |  |  |  |  | 0.029 (0.002) | 0.00 | 0.16 | 0.5 (0.034) | 0.25 | 1.00 |
| Difference |  | 0.025 | 0.064 (0.001) | 0.04 | 1.55 | 0.029 (0.003) | 0.01 | 0.55 | 0.035 (0.002) | 0.03 | 4.54 | 0.545 (0.043) | 0.29 | 1.18 |
| Product |  |  |  |  |  |  |  |  | 0.035 (0.002) | 0.03 | 4.54 | 0.545 (0.043) | 0.29 | 1.18 |
| Difference |  | 0.05 | 0.068 (0.001) | 0.02 | 0.36 | 0.029 (0.004) | -0.01 | -0.24 | 0.039 (0.004) | 0.03 | 2.16 | 0.581 (0.061) | 0.33 | 1.32 |
| Product |  |  |  |  |  |  |  |  | 0.039 (0.004) | 0.03 | 2.16 | 0.581 (0.061) | 0.33 | 1.32 |
| Difference | 0.75 | 0 | 0.051 (0.002) | 0.05 | NA | 0.028 (0.002) | 0.03 | NA | 0.023 (0.001) | 0.02 | NA | 0.447 (0.028) | -0.30 | -0.40 |
| Product |  |  |  |  |  |  |  |  | 0.023 (0.001) | 0.02 | NA | 0.447 (0.028) | -0.30 | -0.40 |
| Difference |  | 0.1 | 0.058 (0.001) | -0.04 | -0.21 | 0.01 (0.003) | -0.02 | -0.61 | 0.048 (0.002) | -0.03 | -0.36 | 0.833 (0.054) | 0.08 | 0.11 |
| Product |  |  |  |  |  |  |  |  | 0.048 (0.002) | -0.03 | -0.36 | 0.833 (0.054) | 0.08 | 0.11 |
| Difference |  | 0.025 | 0.064 (0.001) | 0.04 | 1.54 | -0.01 (0.006) | -0.02 | -2.55 | 0.073 (0.005) | 0.05 | 2.91 | 1.153 (0.091) | 0.40 | 0.54 |
| Product |  |  |  |  |  |  |  |  | 0.073 (0.005) | 0.05 | 2.91 | 1.153 (0.091) | 0.40 | 0.54 |
| Difference |  | 0.05 | 0.068 (0.001) | 0.02 | 0.36 | -0.028 (0.01) | -0.04 | -3.26 | 0.096 (0.009) | 0.06 | 1.57 | 1.416 (0.143) | 0.67 | 0.89 |
| Product |  |  |  |  |  |  |  |  | 0.096 (0.009) | 0.06 | 1.57 | 1.416 (0.143) | 0.67 | 0.89 |

## sTable 7: Estimated effect sizes and size of bias for simulated effect of a continuous mediator explaining the effect between a continuous exposure and common binary outcome using the non-IV difference in coefficients (difference) method and product of coefficients (product) method on the risk difference scale (Simulated N=5000)

| **Mediation method** | **True proportion mediated** | **True total effect** | **Total effect (SD)** | **Size of bias (absolute)** | **Size of bias (relative)** | **Direct effect (SD)** | **Size of bias (absolute)** | **Size of bias (relative)** | **Indirect effect (SD)** | **Size of bias (absolute)** | **Size of bias (relative)** | **Proportion mediated (SD)** | **Size of bias (absolute)** | **Size of bias (relative)** |
| --- | --- | --- | --- | --- | --- | --- | --- | --- | --- | --- | --- | --- | --- | --- |
| Difference | 0 | 0.125 | 0.196 (0.002) | 0.07 | 0.57 | 0.148 (0.003) | 0.02 | 0.19 | 0.048 (0.002) | 0.05 | NA | 0.243 (0.011) | 0.24 | NA |
| Product |  |  |  |  |  |  |  |  | 0.048 (0.002) | 0.05 | NA | 0.243 (0.011) | 0.24 | NA |
| Difference | -0.5 | 0.125 | 0.196 (0.002) | 0.07 | 0.57 | 0.267 (0.003) | 0.08 | 0.43 | -0.071 (0.003) | -0.26 | 4.14 | -0.364 (0.018) | 0.14 | -0.27 |
| Product |  |  |  |  |  |  |  |  | -0.071 (0.003) | -0.26 | 4.14 | -0.364 (0.018) | 0.14 | -0.27 |
| Difference | 0.05 | 0 | 0.157 (0.003) | 0.16 | NA | 0.087 (0.004) | 0.09 | NA | 0.07 (0.002) | 0.07 | NA | 0.445 (0.017) | 0.40 | 7.91 |
| Product |  |  |  |  |  |  |  |  | 0.07 (0.002) | 0.07 | NA | 0.445 (0.017) | 0.40 | 7.91 |
| Difference |  | 0.05 | 0.178 (0.003) | 0.13 | 2.56 | 0.113 (0.004) | 0.07 | 1.38 | 0.065 (0.002) | 0.06 | 25.09 | 0.366 (0.014) | 0.32 | 6.33 |
| Product |  |  |  |  |  |  |  |  | 0.065 (0.002) | 0.06 | 25.09 | 0.366 (0.014) | 0.32 | 6.33 |
| Difference |  | 0.125 | 0.196 (0.002) | 0.07 | 0.57 | 0.137 (0.004) | 0.02 | 0.15 | 0.059 (0.002) | 0.05 | 8.49 | 0.303 (0.014) | 0.25 | 5.05 |
| Product |  |  |  |  |  |  |  |  | 0.059 (0.002) | 0.05 | 8.49 | 0.303 (0.014) | 0.25 | 5.05 |
| Difference |  | 0.25 | 0.21 (0.002) | -0.04 | -0.16 | 0.157 (0.004) | -0.08 | -0.34 | 0.052 (0.003) | 0.04 | 3.19 | 0.25 (0.013) | 0.20 | 4.00 |
| Product |  |  |  |  |  |  |  |  | 0.052 (0.003) | 0.04 | 3.19 | 0.25 (0.013) | 0.20 | 4.00 |
| Difference | 0.25 | 0 | 0.157 (0.003) | 0.16 | NA | 0.087 (0.004) | 0.09 | NA | 0.07 (0.002) | 0.07 | NA | 0.444 (0.017) | 0.19 | 0.78 |
| Product |  |  |  |  |  |  |  |  | 0.07 (0.002) | 0.07 | NA | 0.444 (0.017) | 0.19 | 0.78 |
| Difference |  | 0.05 | 0.178 (0.003) | 0.13 | 2.56 | 0.089 (0.004) | 0.05 | 1.37 | 0.089 (0.003) | 0.08 | 6.12 | 0.5 (0.018) | 0.25 | 1.00 |
| Product |  |  |  |  |  |  |  |  | 0.089 (0.003) | 0.08 | 6.12 | 0.5 (0.018) | 0.25 | 1.00 |
| Difference |  | 0.125 | 0.196 (0.002) | 0.07 | 0.57 | 0.089 (0.005) | 0.00 | -0.05 | 0.107 (0.004) | 0.08 | 2.42 | 0.545 (0.023) | 0.29 | 1.18 |
| Product |  |  |  |  |  |  |  |  | 0.107 (0.004) | 0.08 | 2.42 | 0.545 (0.023) | 0.29 | 1.18 |
| Difference |  | 0.25 | 0.21 (0.002) | -0.04 | -0.16 | 0.087 (0.007) | -0.10 | -0.53 | 0.122 (0.006) | 0.06 | 0.96 | 0.583 (0.03) | 0.33 | 1.33 |
| Product |  |  |  |  |  |  |  |  | 0.122 (0.006) | 0.06 | 0.96 | 0.583 (0.03) | 0.33 | 1.33 |
| Difference | 0.75 | 0 | 0.157 (0.003) | 0.16 | NA | 0.087 (0.004) | 0.09 | NA | 0.07 (0.002) | 0.07 | NA | 0.444 (0.016) | -0.31 | -0.41 |
| Product |  |  |  |  |  |  |  |  | 0.07 (0.002) | 0.07 | NA | 0.444 (0.016) | -0.31 | -0.41 |
| Difference |  | 0.05 | 0.178 (0.003) | 0.13 | 2.56 | 0.03 (0.006) | 0.02 | 1.36 | 0.148 (0.004) | 0.11 | 2.96 | 0.834 (0.03) | 0.08 | 0.11 |
| Product |  |  |  |  |  |  |  |  | 0.148 (0.004) | 0.11 | 2.96 | 0.834 (0.03) | 0.08 | 0.11 |
| Difference |  | 0.125 | 0.196 (0.002) | 0.07 | 0.57 | -0.029 (0.009) | -0.06 | -1.94 | 0.225 (0.008) | 0.13 | 1.40 | 1.149 (0.047) | 0.40 | 0.53 |
| Product |  |  |  |  |  |  |  |  | 0.225 (0.008) | 0.13 | 1.40 | 1.149 (0.047) | 0.40 | 0.53 |
| Difference |  | 0.25 | 0.21 (0.002) | -0.04 | -0.16 | -0.087 (0.015) | -0.15 | -2.39 | 0.297 (0.014) | 0.11 | 0.58 | 1.415 (0.073) | 0.66 | 0.89 |
| Product |  |  |  |  |  |  |  |  | 0.297 (0.014) | 0.11 | 0.58 | 1.415 (0.073) | 0.66 | 0.89 |

## sTable 8: Estimated effect sizes and size of bias for simulated effect of a continuous mediator explaining the effect between a continuous exposure and rare binary outcome using the non-IV difference in coefficients (difference) method and product of coefficients (product) method on the risk difference scale, where simulated total effects are small (Simulated N=5000)

| **Mediation method** | **True proportion mediated** | **True total effect** | **Total effect (SD)** | **Size of bias (absolute)** | **Size of bias (relative)** | **Direct effect (SD)** | **Size of bias (absolute)** | **Size of bias (relative)** | **Indirect effect (SD)** | **Size of bias (absolute)** | **Size of bias (relative)** | **Proportion mediated (SD)** | **Size of bias (absolute)** | **Size of bias (relative)** |
| --- | --- | --- | --- | --- | --- | --- | --- | --- | --- | --- | --- | --- | --- | --- |
| Difference | 0.05 | 0.0005 | 0.051 (0.002) | 0.05 | 101.47 | 0.029 (0.002) | 0.03 | 59.32 | 0.023 (0.001) | 0.02 | 884.28 | 0.441 (0.029) | 0.39 | 7.83 |
| Product |  |  |  |  |  |  |  |  | 0.023 (0.001) | 0.02 | 884.28 | 0.441 (0.029) | 0.39 | 7.83 |
| Difference |  | 0.0025 | 0.053 (0.002) | 0.05 | 20.17 | 0.031 (0.002) | 0.03 | 11.90 | 0.022 (0.001) | 0.02 | 159.28 | 0.422 (0.027) | 0.37 | 7.43 |
| Product |  |  |  |  |  |  |  |  | 0.022 (0.001) | 0.02 | 159.28 | 0.422 (0.027) | 0.37 | 7.43 |
| Difference |  | 0.005 | 0.055 (0.002) | 0.05 | 9.93 | 0.033 (0.002) | 0.03 | 6.80 | 0.022 (0.001) | 0.02 | 85.70 | 0.401 (0.028) | 0.35 | 7.02 |
| Product |  |  |  |  |  |  |  |  | 0.022 (0.001) | 0.02 | 85.70 | 0.401 (0.028) | 0.35 | 7.02 |
| Difference | 0.25 | 0.0005 | 0.051 (0.002) | 0.05 | 101.63 | 0.028 (0.002) | 0.03 | 74.49 | 0.023 (0.001) | 0.02 | 181.05 | 0.449 (0.029) | 0.20 | 0.80 |
| Product |  |  |  |  |  |  |  |  | 0.023 (0.001) | 0.02 | 181.05 | 0.449 (0.029) | 0.20 | 0.80 |
| Difference |  | 0.0025 | 0.053 (0.002) | 0.05 | 20.17 | 0.028 (0.002) | 0.03 | 14.19 | 0.024 (0.001) | 0.02 | 36.10 | 0.462 (0.03) | 0.21 | 0.85 |
| Product |  |  |  |  |  |  |  |  | 0.024 (0.001) | 0.02 | 36.10 | 0.462 (0.03) | 0.21 | 0.85 |
| Difference |  | 0.005 | 0.055 (0.001) | 0.05 | 9.94 | 0.029 (0.002) | 0.03 | 7.54 | 0.026 (0.001) | 0.03 | 20.56 | 0.477 (0.031) | 0.23 | 0.91 |
| Product |  |  |  |  |  |  |  |  | 0.026 (0.001) | 0.03 | 20.56 | 0.477 (0.031) | 0.23 | 0.91 |
| Difference | 0.75 | 0.0005 | 0.051 (0.002) | 0.05 | 101.74 | 0.027 (0.002) | 0.03 | 217.47 | 0.024 (0.001) | 0.02 | 63.83 | 0.469 (0.031) | -0.28 | -0.37 |
| Product |  |  |  |  |  |  |  |  | 0.024 (0.001) | 0.02 | 63.83 | 0.469 (0.031) | -0.28 | -0.37 |
| Difference |  | 0.0025 | 0.053 (0.002) | 0.05 | 20.14 | 0.023 (0.003) | 0.02 | 35.87 | 0.03 (0.002) | 0.03 | 15.57 | 0.565 (0.038) | -0.19 | -0.25 |
| Product |  |  |  |  |  |  |  |  | 0.03 (0.002) | 0.03 | 15.57 | 0.565 (0.038) | -0.19 | -0.25 |
| Difference |  | 0.005 | 0.055 (0.001) | 0.05 | 9.93 | 0.018 (0.003) | 0.02 | 14.37 | 0.037 (0.002) | 0.04 | 9.71 | 0.67 (0.042) | -0.08 | -0.11 |
| Product |  |  |  |  |  |  |  |  | 0.037 (0.002) | 0.04 | 9.71 | 0.67 (0.042) | -0.08 | -0.11 |

## sTable 9: Estimated effect sizes and size of bias for simulated effect of a continuous mediator explaining the effect between a continuous exposure and common binary outcome using the non-IV difference in coefficients (difference) method and product of coefficients (product) method on the risk difference scale, where true total effects are small (Simulated N=5000)

| **Mediation method** | **True proportion mediated** | **True total effect** | **Total effect (SD)** | **Size of bias (absolute)** | **Size of bias (relative)** | **Direct effect (SD)** | **Size of bias (absolute)** | **Size of bias (relative)** | **Indirect effect (SD)** | **Size of bias (absolute)** | **Size of bias (relative)** | **Proportion mediated (SD)** | **Size of bias (absolute)** | **Size of bias (relative)** |
| --- | --- | --- | --- | --- | --- | --- | --- | --- | --- | --- | --- | --- | --- | --- |
| Difference | 0.05 | 0.0025 | 0.158 (0.003) | 0.16 | 62.22 | 0.089 (0.004) | 0.09 | 36.27 | 0.07 (0.002) | 0.07 | 537.24 | 0.44 (0.017) | 0.39 | 7.80 |
| Product |  |  |  |  |  |  |  |  | 0.07 (0.002) | 0.07 | 537.24 | 0.44 (0.017) | 0.39 | 7.80 |
| Difference |  | 0.0125 | 0.163 (0.003) | 0.15 | 12.05 | 0.095 (0.004) | 0.08 | 6.96 | 0.069 (0.002) | 0.06 | 90.70 | 0.42 (0.016) | 0.37 | 7.41 |
| Product |  |  |  |  |  |  |  |  | 0.069 (0.002) | 0.06 | 90.70 | 0.42 (0.016) | 0.37 | 7.41 |
| Difference |  | 0.025 | 0.169 (0.003) | 0.14 | 5.74 | 0.101 (0.004) | 0.08 | 3.26 | 0.067 (0.002) | 0.04 | 34.97 | 0.4 (0.015) | 0.35 | 7.01 |
| Product |  |  |  |  |  |  |  |  | 0.067 (0.002) | 0.04 | 34.97 | 0.4 (0.015) | 0.35 | 7.01 |
| Difference | 0.25 | 0.0025 | 0.158 (0.003) | 0.16 | 62.22 | 0.087 (0.004) | 0.09 | 45.56 | 0.071 (0.002) | 0.07 | 110.19 | 0.448 (0.016) | 0.20 | 0.79 |
| Product |  |  |  |  |  |  |  |  | 0.071 (0.002) | 0.07 | 110.19 | 0.448 (0.016) | 0.20 | 0.79 |
| Difference |  | 0.0125 | 0.163 (0.003) | 0.15 | 12.06 | 0.088 (0.004) | 0.08 | 8.38 | 0.075 (0.002) | 0.07 | 21.08 | 0.461 (0.017) | 0.21 | 0.85 |
| Product |  |  |  |  |  |  |  |  | 0.075 (0.002) | 0.07 | 21.08 | 0.461 (0.017) | 0.21 | 0.85 |
| Difference |  | 0.025 | 0.169 (0.003) | 0.14 | 5.75 | 0.088 (0.004) | 0.07 | 3.72 | 0.08 (0.002) | 0.06 | 9.84 | 0.476 (0.017) | 0.23 | 0.90 |
| Product |  |  |  |  |  |  |  |  | 0.08 (0.002) | 0.06 | 9.84 | 0.476 (0.017) | 0.23 | 0.90 |
| Difference | 0.75 | 0.0025 | 0.158 (0.003) | 0.16 | 62.22 | 0.084 (0.004) | 0.08 | 133.04 | 0.074 (0.002) | 0.07 | 39.28 | 0.47 (0.017) | -0.28 | -0.37 |
| Product |  |  |  |  |  |  |  |  | 0.074 (0.002) | 0.07 | 39.28 | 0.47 (0.017) | -0.28 | -0.37 |
| Difference |  | 0.0125 | 0.163 (0.003) | 0.15 | 12.05 | 0.071 (0.004) | 0.07 | 21.75 | 0.092 (0.003) | 0.09 | 9.48 | 0.564 (0.02) | -0.19 | -0.25 |
| Product |  |  |  |  |  |  |  |  | 0.092 (0.003) | 0.09 | 9.48 | 0.564 (0.02) | -0.19 | -0.25 |
| Difference |  | 0.025 | 0.169 (0.003) | 0.14 | 5.75 | 0.056 (0.005) | 0.05 | 8.01 | 0.112 (0.003) | 0.11 | 5.66 | 0.666 (0.023) | -0.08 | -0.11 |
| Product |  |  |  |  |  |  |  |  | 0.112 (0.003) | 0.11 | 5.66 | 0.666 (0.023) | -0.08 | -0.11 |

## sTable 10: Estimated effect sizes and size of bias for simulated effect of a continuous mediator explaining the effect between a continuous exposure and a rare binary outcome using the Mendelian randomisation methods multivariable Mendelian randomisation (MVMR) and two-step Mendelian randomisation (TSMR) on the risk difference scale (Simulated N=5000)

| **Mediation method** | **True proportion mediated** | **True total effect** | **Total effect (SD)** | **Size of bias (absolute)** | **Size of bias (relative)** | **Direct effect (SD)** | **Size of bias (absolute)** | **Size of bias (relative)** | **Indirect effect (SD)** | **Size of bias (absolute)** | **Size of bias (relative)** | **Proportion mediated (SD)** | **Size of bias (absolute)** | **Size of bias (relative)** |
| --- | --- | --- | --- | --- | --- | --- | --- | --- | --- | --- | --- | --- | --- | --- |
| MVMR | 0 | 0.025 | 0.029 (0.003) | 0.00 | 0.15 | 0.029 (0.003) | 0.00 | 0.15 | 0 (0) | 0.00 | NA | -0.001 (0.008) | 0.00 | NA |
| TSMR |  |  |  |  |  |  |  |  | 0 (0) | 0.00 | NA | 0 (-0.001) | 0.00 | NA |
| MVMR | -0.5 | 0.025 | 0.029 (0.003) | 0.00 | 0.15 | 0.043 (0.004) | 0.01 | 0.15 | -0.014 (0.003) | 0.00 | 0.16 | -0.509 (0.133) | -0.01 | 0.02 |
| TSMR |  |  |  |  |  |  |  |  | -0.014 (0.003) | 0.00 | 0.16 | -0.509 (0.133) | -0.01 | 0.02 |
| MVMR | 0.05 | 0 | 0 (0.003) | 0.00 | NA | 0 (0.003) | 0.00 | NA | 0 (0) | 0.00 | NA | -0.038 (2.344) | -0.09 | -1.77 |
| TSMR |  |  |  |  |  |  |  |  | 0 (0) | 0.00 | NA | 0 (-0.038) | -0.09 | -1.77 |
| MVMR |  | 0.1 | 0.014 (0.003) | -0.09 | -0.86 | 0.014 (0.003) | -0.08 | -0.86 | 0.001 (0) | 0.00 | -0.85 | 0.051 (0.023) | 0.00 | 0.02 |
| TSMR |  |  |  |  |  |  |  |  | 0.001 (0) | 0.00 | -0.85 | 0 (0.051) | 0.00 | 0.02 |
| MVMR |  | 0.025 | 0.029 (0.003) | 0.00 | 0.15 | 0.027 (0.003) | 0.00 | 0.15 | 0.001 (0) | 0.00 | 0.15 | 0.05 (0.015) | 0.00 | 0.01 |
| TSMR |  |  |  |  |  |  |  |  | 0.001 (0) | 0.00 | 0.15 | 0 (0.05) | 0.00 | 0.01 |
| MVMR |  | 0.05 | 0.043 (0.003) | -0.01 | -0.15 | 0.04 (0.003) | -0.01 | -0.15 | 0.002 (0.001) | 0.00 | -0.14 | 0.051 (0.017) | 0.00 | 0.02 |
| TSMR |  |  |  |  |  |  |  |  | 0.002 (0.001) | 0.00 | -0.14 | 0.001 (0.051) | 0.00 | 0.02 |
| MVMR | 0.25 | 0 | 0 (0.003) | 0.00 | NA | 0 (0.003) | 0.00 | NA | 0 (0) | 0.00 | NA | -0.08 (3.313) | -0.33 | -1.32 |
| TSMR |  |  |  |  |  |  |  |  | 0 (0) | 0.00 | NA | 0 (-0.08) | -0.33 | -1.32 |
| MVMR |  | 0.1 | 0.014 (0.003) | -0.09 | -0.43 | 0.011 (0.003) | -0.06 | -0.86 | 0.004 (0.001) | -0.02 | -0.86 | 0.26 (0.078) | 0.01 | 0.04 |
| TSMR |  |  |  |  |  |  |  |  | 0.004 (0.001) | -0.02 | -0.86 | 0.001 (0.26) | 0.01 | 0.04 |
| MVMR |  | 0.025 | 0.029 (0.003) | 0.00 | 0.16 | 0.022 (0.003) | 0.00 | 0.17 | 0.007 (0.002) | 0.00 | 0.15 | 0.25 (0.066) | 0.00 | 0.00 |
| TSMR |  |  |  |  |  |  |  |  | 0.007 (0.002) | 0.00 | 0.15 | 0.002 (0.25) | 0.00 | 0.00 |
| MVMR |  | 0.05 | 0.043 (0.003) | -0.01 | -0.15 | 0.032 (0.004) | -0.01 | -0.14 | 0.011 (0.004) | 0.00 | -0.16 | 0.248 (0.085) | 0.00 | -0.01 |
| TSMR |  |  |  |  |  |  |  |  | 0.011 (0.004) | 0.00 | -0.16 | 0.004 (0.248) | 0.00 | -0.01 |
| MVMR | 0.75 | 0 | 0 (0.003) | 0.00 | NA | 0 (0.003) | 0.00 | NA | 0 (0) | 0.00 |  | 0.027 (2.198) | -0.72 | -0.96 |
| TSMR |  |  |  |  |  |  |  |  | 0 (0) | 0.00 |  | 0 (0.027) | -0.72 | -0.96 |
| MVMR |  | 0.1 | 0.014 (0.003) | -0.09 | -0.43 | 0.004 (0.004) | -0.02 | -0.85 | 0.011 (0.002) | -0.06 | -0.86 | 0.782 (0.237) | 0.03 | 0.04 |
| TSMR |  |  |  |  |  |  |  |  | 0.011 (0.002) | -0.06 | -0.86 | 0.002 (0.782) | 0.03 | 0.04 |
| MVMR |  | 0.025 | 0.029 (0.003) | 0.00 | 0.16 | 0.007 (0.006) | 0.00 | 0.16 | 0.022 (0.005) | 0.00 | 0.16 | 0.757 (0.202) | 0.01 | 0.01 |
| TSMR |  |  |  |  |  |  |  |  | 0.022 (0.005) | 0.00 | 0.16 | 0.005 (0.757) | 0.01 | 0.01 |
| MVMR |  | 0.05 | 0.043 (0.003) | -0.01 | -0.15 | 0.011 (0.011) | 0.00 | -0.15 | 0.032 (0.01) | -0.01 | -0.15 | 0.753 (0.247) | 0.00 | 0.00 |
| TSMR |  |  |  |  |  |  |  |  | 0.032 (0.01) | -0.01 | -0.15 | 0.01 (0.753) | 0.00 | 0.00 |

Total effect = estimated using univariate Mendelian randomisation; direct effect = estimated using multivariable Mendelian randomisation controlling for both exposure and mediator

## sTable 11: Estimated effect sizes and size of bias for simulated effect of a continuous mediator explaining the effect between a continuous exposure and a common binary outcome using the Mendelian randomisation methods multivariable Mendelian randomisation (MVMR) and two-step Mendelian randomisation (TSMR) on the risk difference scale (Simulated N=5000)

| **Mediation method** | **True proportion mediated** | **True total effect** | **Total effect (SD)** | **Size of bias (absolute)** | **Size of bias (relative)** | **Direct effect (SD)** | **Size of bias (absolute)** | **Size of bias (relative)** | **Indirect effect (SD)** | **Size of bias (absolute)** | **Size of bias (relative)** | **Proportion mediated (SD)** | **Size of bias (absolute)** | **Size of bias (relative)** |
| --- | --- | --- | --- | --- | --- | --- | --- | --- | --- | --- | --- | --- | --- | --- |
| MVMR | 0 | 0.125 | 0.089 (0.005) | -0.04 | -0.29 | 0.089 (0.005) | -0.04 | -0.29 | 0 (0.001) | 0.00 | NA | -0.001 (0.008) | 0.00 | NA |
| TSMR |  |  |  |  |  |  |  |  | 0 (0.001) | 0.00 | NA | -0.001 (0.008) | 0.00 | NA |
| MVMR | -0.5 | 0.125 | 0.089 (0.005) | -0.04 | -0.29 | 0.133 (0.007) | -0.05 | -0.29 | -0.045 (0.006) | 0.02 | -0.29 | -0.503 (0.079) | 0.00 | 0.01 |
| TSMR |  |  |  |  |  |  |  |  | -0.045 (0.006) | 0.02 | -0.29 | -0.503 (0.079) | 0.00 | 0.01 |
| MVMR | 0.05 | 0 | 0 (0.006) | 0.00 | NA | 0 (0.006) | 0.00 | NA | 0 (0.001) | 0.00 | NA | -1.207 (38.778) | -1.26 | -25.14 |
| TSMR |  |  |  |  |  |  |  |  | 0 (0.001) | 0.00 | NA | -1.207 (38.778) | -1.26 | -25.14 |
| MVMR |  | 0.05 | 0.045 (0.006) | -0.01 | -0.10 | 0.043 (0.005) | 0.00 | -0.10 | 0.002 (0.001) | 0.00 | -0.10 | 0.05 (0.019) | 0.00 | -0.01 |
| TSMR |  |  |  |  |  |  |  |  | 0.002 (0.001) | 0.00 | -0.10 | 0.05 (0.019) | 0.00 | -0.01 |
| MVMR |  | 0.125 | 0.089 (0.005) | -0.04 | -0.29 | 0.085 (0.005) | -0.03 | -0.29 | 0.004 (0.001) | 0.00 | -0.29 | 0.05 (0.01) | 0.00 | -0.01 |
| TSMR |  |  |  |  |  |  |  |  | 0.004 (0.001) | 0.00 | -0.29 | 0.05 (0.01) | 0.00 | -0.01 |
| MVMR |  | 0.25 | 0.131 (0.004) | -0.12 | -0.48 | 0.125 (0.004) | -0.11 | -0.48 | 0.007 (0.001) | -0.01 | -0.48 | 0.05 (0.009) | 0.00 | 0.00 |
| TSMR |  |  |  |  |  |  |  |  | 0.007 (0.001) | -0.01 | -0.48 | 0.05 (0.009) | 0.00 | 0.00 |
| MVMR | 0.25 | 0 | 0 (0.006) | 0.00 | NA | 0 (0.006) | 0.00 | NA | 0 (0.001) | 0.00 | NA | 1.384 (49.314) | 1.13 | 4.54 |
| TSMR |  |  |  |  |  |  |  |  | 0 (0.001) | 0.00 | NA | 1.384 (49.314) | 1.13 | 4.54 |
| MVMR |  | 0.05 | 0.044 (0.005) | -0.01 | -0.12 | 0.033 (0.005) | 0.00 | -0.12 | 0.011 (0.002) | 0.00 | -0.12 | 0.252 (0.04) | 0.00 | 0.01 |
| TSMR |  |  |  |  |  |  |  |  | 0.011 (0.002) | 0.00 | -0.12 | 0.252 (0.04) | 0.00 | 0.01 |
| MVMR |  | 0.125 | 0.089 (0.005) | -0.04 | -0.29 | 0.067 (0.006) | -0.03 | -0.28 | 0.022 (0.003) | -0.01 | -0.29 | 0.248 (0.037) | 0.00 | -0.01 |
| TSMR |  |  |  |  |  |  |  |  | 0.022 (0.003) | -0.01 | -0.29 | 0.248 (0.037) | 0.00 | -0.01 |
| MVMR |  | 0.25 | 0.131 (0.004) | -0.12 | -0.48 | 0.098 (0.007) | -0.09 | -0.48 | 0.033 (0.006) | -0.03 | -0.47 | 0.25 (0.044) | 0.00 | 0.00 |
| TSMR |  |  |  |  |  |  |  |  | 0.033 (0.006) | -0.03 | -0.47 | 0.25 (0.044) | 0.00 | 0.00 |
| MVMR | 0.75 | 0 | 0 (0.006) | 0.00 | NA | 0 (0.005) | 0.00 | NA | 0 (0.001) | 0.00 | NA | -0.065 (9.881) | -0.81 | -1.09 |
| TSMR |  |  |  |  |  |  |  |  | 0 (0.001) | 0.00 | NA | -0.065 (9.881) | -0.81 | -1.09 |
| MVMR |  | 0.05 | 0.044 (0.006) | -0.01 | -0.11 | 0.011 (0.006) | 0.00 | -0.12 | 0.033 (0.004) | 0.00 | -0.11 | 0.764 (0.13) | 0.01 | 0.02 |
| TSMR |  |  |  |  |  |  |  |  | 0.033 (0.004) | 0.00 | -0.11 | 0.764 (0.13) | 0.01 | 0.02 |
| MVMR |  | 0.125 | 0.089 (0.005) | -0.04 | -0.29 | 0.023 (0.01) | -0.01 | -0.26 | 0.066 (0.009) | -0.03 | -0.29 | 0.744 (0.109) | -0.01 | -0.01 |
| TSMR |  |  |  |  |  |  |  |  | 0.066 (0.009) | -0.03 | -0.29 | 0.744 (0.109) | -0.01 | -0.01 |
| MVMR |  | 0.25 | 0.131 (0.004) | -0.12 | -0.48 | 0.033 (0.017) | -0.03 | -0.47 | 0.098 (0.017) | -0.09 | -0.48 | 0.748 (0.131) | 0.00 | 0.00 |
| TSMR |  |  |  |  |  |  |  |  | 0.098 (0.017) | -0.09 | -0.48 | 0.748 (0.131) | 0.00 | 0.00 |

Total effect = estimated using univariate Mendelian randomisation; direct effect = estimated using multivariable Mendelian randomisation controlling for both exposure and mediator

## sTable 12: Estimated effect sizes and size of bias for simulated effect of a continuous mediator explaining the effect between a continuous exposure and rare binary outcome using the Mendelian randomisation methods multivariable Mendelian randomisation (MVMR) and two-step Mendelian randomisation (TSMR) on the risk difference scale, where simulated total effects are small (Simulated N=5000)

| **Mediation method** | **True proportion mediated** | **True total effect** | **Total effect (SD)** | **Size of bias (absolute)** | **Size of bias (relative)** | **Direct effect (SD)** | **Size of bias (absolute)** | **Size of bias (relative)** | **Indirect effect (SD)** | **Size of bias (absolute)** | **Size of bias (relative)** | **Proportion mediated (SD)** | **Size of bias (absolute)** | **Size of bias (relative)** |
| --- | --- | --- | --- | --- | --- | --- | --- | --- | --- | --- | --- | --- | --- | --- |
| MVMR | 0.05 | 0.0005 | 0.001 (0.003) | 0.00 | 0.39 | 0.001 (0.003) | 0.00 | 0.38 | 0 (0) | 0.00 | 0.56 | -0.021 (5.936) | -0.07 | -1.42 |
| TSMR |  |  |  |  |  |  |  |  | 0 (0) | 0.00 | 0.56 | -0.021 (5.936) | -0.07 | -1.42 |
| MVMR |  | 0.0025 | 0.004 (0.003) | 0.00 | 0.64 | 0.004 (0.003) | 0.00 | 0.64 | 0 (0) | 0.00 | 0.71 | 0.05 (0.682) | 0.00 | -0.01 |
| TSMR |  |  |  |  |  |  |  |  | 0 (0) | 0.00 | 0.71 | 0.05 (0.682) | 0.00 | -0.01 |
| MVMR |  | 0.005 | 0.008 (0.003) | 0.01 | 1.45 | 0.007 (0.003) | 0.01 | 1.45 | 0 (0) | 0.00 | 1.45 | 0.05 (0.169) | 0.00 | -0.01 |
| TSMR |  |  |  |  |  |  |  |  | 0 (0) | 0.00 | 1.45 | 0.05 (0.169) | 0.00 | -0.01 |
| MVMR | 0.25 | 0.0005 | 0.001 (0.003) | 0.00 | 0.70 | 0.001 (0.003) | 0.00 | 0.71 | 0 (0) | 0.00 | 0.65 | 0.055 (1.544) | -0.20 | -0.78 |
| TSMR |  |  |  |  |  |  |  |  | 0 (0) | 0.00 | 0.65 | 0.055 (1.544) | -0.20 | -0.78 |
| MVMR |  | 0.0025 | 0.004 (0.003) | 0.00 | 0.61 | 0.003 (0.003) | 0.00 | 0.61 | 0.001 (0) | 0.00 | 0.62 | 1.169 (30.416) | 0.92 | 3.68 |
| TSMR |  |  |  |  |  |  |  |  | 0.001 (0) | 0.00 | 0.62 | 1.169 (30.416) | 0.92 | 3.68 |
| MVMR |  | 0.005 | 0.008 (0.003) | 0.01 | 1.44 | 0.006 (0.003) | 0.01 | 1.43 | 0.002 (0) | 0.00 | 1.45 | 0.275 (0.864) | 0.02 | 0.10 |
| TSMR |  |  |  |  |  |  |  |  | 0.002 (0) | 0.00 | 1.45 | 0.275 (0.864) | 0.02 | 0.10 |
| MVMR | 0.75 | 0.0005 | 0.001 (0.003) | 0.00 | 0.96 | 0 (0.003) | 0.00 | 1.86 | 0.001 (0) | 0.00 | 0.66 | -0.061 (7.019) | -0.81 | -1.08 |
| TSMR |  |  |  |  |  |  |  |  | 0.001 (0) | 0.00 | 0.66 | -0.061 (7.019) | -0.81 | -1.08 |
| MVMR |  | 0.0025 | 0.004 (0.003) | 0.00 | 0.64 | 0.001 (0.003) | 0.00 | 0.67 | 0.003 (0.001) | 0.00 | 0.63 | 0.492 (18.682) | -0.26 | -0.34 |
| TSMR |  |  |  |  |  |  |  |  | 0.003 (0.001) | 0.00 | 0.63 | 0.492 (18.682) | -0.26 | -0.34 |
| MVMR |  | 0.005 | 0.008 (0.003) | 0.01 | 1.43 | 0.002 (0.003) | 0.00 | 1.33 | 0.006 (0.001) | 0.01 | 1.47 | 1.047 (2.14) | 0.30 | 0.40 |
| TSMR |  |  |  |  |  |  |  |  | 0.006 (0.001) | 0.01 | 1.47 | 1.047 (2.14) | 0.30 | 0.40 |

Total effect = estimated using univariate Mendelian randomisation; direct effect = estimated using multivariable Mendelian randomisation controlling for both exposure and mediator

## sTable 13: Estimated effect sizes and size of bias for simulated effect of a continuous mediator explaining the effect between a continuous exposure and common binary outcome using the Mendelian randomisation methods multivariable Mendelian randomisation (MVMR) and two-step Mendelian randomisation (TSMR) on the risk difference scale, where simulated total effects are small (Simulated N=5000)

| **Mediation method** | **True proportion mediated** | **True total effect** | **Total effect (SD)** | **Size of bias (absolute)** | **Size of bias (relative)** | **Direct effect (SD)** | **Size of bias (absolute)** | **Size of bias (relative)** | **Indirect effect (SD)** | **Size of bias (absolute)** | **Size of bias (relative)** | **Proportion mediated (SD)** | **Size of bias (absolute)** | **Size of bias (relative)** |
| --- | --- | --- | --- | --- | --- | --- | --- | --- | --- | --- | --- | --- | --- | --- |
| MVMR | 0.05 | 0.0025 | 0.002 (0.006) | 0.00 | -0.02 | 0.002 (0.006) | 0.00 | -0.02 | 0 (0.001) | 0.00 | -0.05 | 0.115 (2.813) | 0.07 | 1.31 |
| TSMR |  |  |  |  |  |  |  |  | 0 (0.001) | 0.00 | -0.05 | 0.115 (2.813) | 0.07 | 1.31 |
| MVMR |  | 0.0125 | 0.013 (0.006) | 0.00 | 0.02 | 0.012 (0.006) | 0.00 | 0.02 | 0.001 (0.001) | 0.00 | 0.04 | 0.047 (0.533) | 0.00 | -0.06 |
| TSMR |  |  |  |  |  |  |  |  | 0.001 (0.001) | 0.00 | 0.04 | 0.047 (0.533) | 0.00 | -0.06 |
| MVMR |  | 0.025 | 0.024 (0.006) | 0.00 | -0.04 | 0.023 (0.005) | 0.00 | -0.04 | 0.001 (0.001) | 0.00 | -0.05 | 0.046 (0.044) | 0.00 | -0.08 |
| TSMR |  |  |  |  |  |  |  |  | 0.001 (0.001) | 0.00 | -0.05 | 0.046 (0.044) | 0.00 | -0.08 |
| MVMR | 0.25 | 0.0025 | 0.003 (0.006) | 0.00 | 0.04 | 0.002 (0.005) | 0.00 | 0.05 | 0.001 (0.001) | 0.00 | 0.01 | 0.243 (5.842) | -0.01 | -0.03 |
| TSMR |  |  |  |  |  |  |  |  | 0.001 (0.001) | 0.00 | 0.01 | 0.243 (5.842) | -0.01 | -0.03 |
| MVMR |  | 0.0125 | 0.013 (0.006) | 0.00 | 0.02 | 0.01 (0.006) | 0.00 | 0.02 | 0.003 (0.001) | 0.00 | 0.00 | 0.287 (1.014) | 0.04 | 0.15 |
| TSMR |  |  |  |  |  |  |  |  | 0.003 (0.001) | 0.00 | 0.00 | 0.287 (1.014) | 0.04 | 0.15 |
| MVMR |  | 0.025 | 0.024 (0.006) | 0.00 | -0.04 | 0.018 (0.005) | 0.00 | -0.04 | 0.006 (0.001) | 0.00 | -0.05 | 0.26 (0.075) | 0.01 | 0.04 |
| TSMR |  |  |  |  |  |  |  |  | 0.006 (0.001) | 0.00 | -0.05 | 0.26 (0.075) | 0.01 | 0.04 |
| MVMR | 0.75 | 0.0025 | 0.003 (0.006) | 0.00 | 0.01 | 0.001 (0.006) | 0.00 | -0.03 | 0.002 (0.001) | 0.00 | 0.02 | 1.458 (28.614) | 0.71 | 0.94 |
| TSMR |  |  |  |  |  |  |  |  | 0.002 (0.001) | 0.00 | 0.02 | 1.458 (28.614) | 0.71 | 0.94 |
| MVMR |  | 0.0125 | 0.013 (0.006) | 0.00 | 0.02 | 0.003 (0.006) | 0.00 | 0.06 | 0.009 (0.001) | 0.00 | 0.01 | 0.877 (4.495) | 0.13 | 0.17 |
| TSMR |  |  |  |  |  |  |  |  | 0.009 (0.001) | 0.00 | 0.01 | 0.877 (4.495) | 0.13 | 0.17 |
| MVMR |  | 0.025 | 0.024 (0.006) | 0.00 | -0.04 | 0.006 (0.006) | 0.00 | -0.02 | 0.018 (0.002) | 0.00 | -0.04 | 0.792 (0.231) | 0.04 | 0.06 |
| TSMR |  |  |  |  |  |  |  |  | 0.018 (0.002) | 0.00 | -0.04 | 0.792 (0.231) | 0.04 | 0.06 |

Total effect = estimated using univariate Mendelian randomisation; direct effect = estimated using multivariable Mendelian randomisation controlling for both exposure and mediator

## sTable 14: Estimated effect sizes and size of bias for simulated effect of a continuous mediator explaining the effect between a continuous exposure and a rare binary outcome using the Mendelian randomisation methods multivariable Mendelian randomisation (MVMR) and two-step Mendelian randomisation (TSMR) on the log odds ratio scale (Simulated N=5000)

| **Mediation method** | **True proportion mediated** | **True total effect** | **Total effect (SD)** | **Size of bias (absolute)** | **Size of bias (relative)** | **Direct effect (SD)** | **Size of bias (absolute)** | **Size of bias (relative)** | **Indirect effect (SD)** | **Size of bias (absolute)** | **Size of bias (relative)** | **Proportion mediated (SD)** | **Size of bias (absolute)** | **Size of bias (relative)** |
| --- | --- | --- | --- | --- | --- | --- | --- | --- | --- | --- | --- | --- | --- | --- |
| MVMR | 0 | 0.5 | 0.617 (0.063) | 0.12 | 0.23 | 0.62 (0.062) | 0.12 | 0.24 | -0.003 (0.006) | 0.00 | NA | -0.004 (0.01) | 0.00 | NA |
| TSMR |  |  |  |  |  |  |  |  | 0 (0.005) | 0.00 | NA | -0.001 (0.008) | 0.00 | NA |
| MVMR | 0.05 | 0 | -0.003 (0.063) | 0.00 | NA | -0.003 (0.062) | 0.00 | NA | 0 (0.007) | 0.00 | NA | -0.049 (2.294) | -0.10 | -1.99 |
| TSMR |  |  |  |  |  |  |  |  | 0 (0.007) | 0.00 | NA | -0.039 (2.356) | -0.09 | -1.78 |
| MVMR |  | 0.2 | 0.306 (0.061) | 0.11 | 0.53 | 0.292 (0.06) | 0.10 | 0.54 | 0.014 (0.007) | 0.00 | 0.39 | 0.046 (0.024) | 0.00 | -0.08 |
| TSMR |  |  |  |  |  |  |  |  | 0.016 (0.007) | 0.01 | 0.56 | 0.051 (0.023) | 0.00 | 0.03 |
| MVMR |  | 0.5 | 0.621 (0.065) | 0.12 | 0.24 | 0.592 (0.064) | 0.12 | 0.25 | 0.028 (0.009) | 0.00 | 0.14 | 0.046 (0.014) | 0.00 | -0.08 |
| TSMR |  |  |  |  |  |  |  |  | 0.031 (0.01) | 0.01 | 0.25 | 0.051 (0.016) | 0.00 | 0.01 |
| MVMR |  | 1 | 0.946 (0.065) | -0.05 | -0.05 | 0.901 (0.066) | -0.05 | -0.05 | 0.045 (0.015) | -0.01 | -0.10 | 0.048 (0.016) | 0.00 | -0.05 |
| TSMR |  |  |  |  |  |  |  |  | 0.048 (0.016) | 0.00 | -0.04 | 0.051 (0.017) | 0.00 | 0.02 |
| MVMR | 0.25 | 0 | 0 (0.063) | 0.00 | NA | 0 (0.061) | 0.00 | NA | 0 (0.007) | 0.00 | NA | -0.048 (3.005) | -0.30 | -1.19 |
| TSMR |  |  |  |  |  |  |  |  | 0 (0.007) | 0.00 | NA | -0.08 (3.316) | -0.33 | -1.32 |
| MVMR |  | 0.2 | 0.305 (0.062) | 0.10 | 0.52 | 0.23 (0.062) | 0.08 | 0.53 | 0.075 (0.016) | 0.02 | 0.50 | 0.256 (0.078) | 0.01 | 0.02 |
| TSMR |  |  |  |  |  |  |  |  | 0.077 (0.017) | 0.03 | 0.53 | 0.261 (0.079) | 0.01 | 0.05 |
| MVMR |  | 0.5 | 0.625 (0.065) | 0.12 | 0.25 | 0.472 (0.073) | 0.10 | 0.26 | 0.153 (0.037) | 0.03 | 0.22 | 0.247 (0.065) | 0.00 | -0.01 |
| TSMR |  |  |  |  |  |  |  |  | 0.156 (0.038) | 0.03 | 0.25 | 0.251 (0.067) | 0.00 | 0.01 |
| MVMR |  | 1 | 0.947 (0.067) | -0.05 | -0.05 | 0.715 (0.102) | -0.04 | -0.05 | 0.232 (0.077) | -0.02 | -0.07 | 0.246 (0.083) | 0.00 | -0.02 |
| TSMR |  |  |  |  |  |  |  |  | 0.235 (0.078) | -0.02 | -0.06 | 0.249 (0.085) | 0.00 | 0.00 |
| MVMR | 0.75 | 0 | -0.003 (0.065) | 0.00 | NA | -0.003 (0.063) | 0.00 | NA | 0 (0.007) | 0.00 | NA | 0.042 (2.245) | -0.71 | -0.94 |
| TSMR |  |  |  |  |  |  |  |  | 0 (0.007) | 0.00 | NA | 0.028 (2.217) | -0.72 | -0.96 |
| MVMR |  | 0.2 | 0.304 (0.063) | 0.10 | 0.52 | 0.077 (0.075) | 0.03 | 0.55 | 0.227 (0.045) | 0.08 | 0.51 | 0.781 (0.237) | 0.03 | 0.04 |
| TSMR |  |  |  |  |  |  |  |  | 0.229 (0.046) | 0.08 | 0.52 | 0.786 (0.239) | 0.04 | 0.05 |
| MVMR |  | 0.5 | 0.622 (0.061) | 0.12 | 0.24 | 0.156 (0.13) | 0.03 | 0.25 | 0.465 (0.115) | 0.09 | 0.24 | 0.756 (0.202) | 0.01 | 0.01 |
| TSMR |  |  |  |  |  |  |  |  | 0.468 (0.116) | 0.09 | 0.25 | 0.761 (0.204) | 0.01 | 0.01 |
| MVMR |  | 1 | 0.945 (0.064) | -0.05 | -0.05 | 0.234 (0.235) | -0.02 | -0.06 | 0.711 (0.227) | -0.04 | -0.05 | 0.756 (0.248) | 0.01 | 0.01 |
| TSMR |  |  |  |  |  |  |  |  | 0.714 (0.229) | -0.04 | -0.05 | 0.759 (0.25) | 0.01 | 0.01 |

Total effect = estimated using univariate Mendelian randomisation; direct effect = estimated using multivariable Mendelian randomisation controlling for both exposure and mediator

## sTable 15: Estimated effect sizes and size of bias for simulated effect of a continuous mediator explaining the effect between a continuous exposure and a common binary outcome using the Mendelian randomisation methods multivariable Mendelian randomisation (MVMR) and two-step Mendelian randomisation (TSMR) on the log odds ratio scale (Simulated N=5000)

| **Mediation method** | **True proportion mediated** | **True total effect** | **Total effect (SD)** | **Size of bias (absolute)** | **Size of bias (relative)** | **Direct effect (SD)** | **Size of bias (absolute)** | **Size of bias (relative)** | **Indirect effect (SD)** | **Size of bias (absolute)** | **Size of bias (relative)** | **Proportion mediated (SD)** | **Size of bias (absolute)** | **Size of bias (relative)** |
| --- | --- | --- | --- | --- | --- | --- | --- | --- | --- | --- | --- | --- | --- | --- |
| MVMR | 0 | 0.5 | 0.496 (0.032) | 0.00 | -0.01 | 0.5 (0.03) | 0.00 | 0.00 | -0.004 (0.004) | 0.00 | NA | -0.008 (0.009) | -0.01 | -0.02 |
| TSMR |  |  |  |  |  |  |  |  | 0 (0.004) | 0.00 | NA | -0.001 (0.008) | 0.00 | 0.00 |
| MVMR | 0.05 | 0 | -0.001 (0.033) | 0.00 | NA | -0.001 (0.03) | 0.00 | NA | 0 (0.005) | 0.00 | NA | -1.244 (38.267) | -1.29 | NA |
| TSMR |  |  |  |  |  |  |  |  | 0 (0.006) | 0.00 | NA | -1.226 (39.475) | -1.28 | NA |
| MVMR |  | 0.2 | 0.242 (0.031) | 0.04 | 0.21 | 0.232 (0.029) | 0.04 | 0.22 | 0.01 (0.005) | 0.00 | -0.05 | 0.039 (0.019) | -0.01 | -0.06 |
| TSMR |  |  |  |  |  |  |  |  | 0.012 (0.005) | 0.00 | 0.23 | 0.05 (0.019) | 0.00 | 0.00 |
| MVMR |  | 0.5 | 0.497 (0.03) | 0.00 | -0.01 | 0.476 (0.029) | 0.00 | 0.00 | 0.021 (0.005) | 0.00 | -0.16 | 0.042 (0.009) | -0.01 | -0.02 |
| TSMR |  |  |  |  |  |  |  |  | 0.025 (0.006) | 0.00 | 0.00 | 0.05 (0.01) | 0.00 | 0.00 |
| MVMR |  | 1 | 0.775 (0.032) | -0.23 | -0.23 | 0.74 (0.032) | -0.21 | -0.22 | 0.035 (0.006) | -0.01 | -0.30 | 0.045 (0.008) | 0.00 | 0.00 |
| TSMR |  |  |  |  |  |  |  |  | 0.039 (0.007) | -0.01 | -0.22 | 0.05 (0.009) | 0.00 | 0.00 |
| MVMR | 0.25 | 0 | 0.001 (0.034) | 0.00 | NA | 0.001 (0.031) | 0.00 | NA | 0 (0.006) | 0.00 | NA | 1.345 (48.216) | 1.10 | NA |
| TSMR |  |  |  |  |  |  |  |  | 0 (0.006) | 0.00 | NA | 1.423 (50.673) | 1.17 | NA |
| MVMR |  | 0.2 | 0.238 (0.03) | 0.04 | 0.19 | 0.181 (0.029) | 0.03 | 0.21 | 0.058 (0.008) | 0.01 | 0.15 | 0.244 (0.039) | -0.01 | -0.04 |
| TSMR |  |  |  |  |  |  |  |  | 0.06 (0.008) | 0.01 | 0.20 | 0.255 (0.041) | 0.01 | 0.04 |
| MVMR |  | 0.5 | 0.498 (0.031) | 0.00 | 0.00 | 0.377 (0.034) | 0.00 | 0.01 | 0.12 (0.017) | 0.00 | -0.04 | 0.243 (0.036) | -0.01 | -0.02 |
| TSMR |  |  |  |  |  |  |  |  | 0.124 (0.018) | 0.00 | -0.01 | 0.25 (0.038) | 0.00 | 0.00 |
| MVMR |  | 1 | 0.775 (0.032) | -0.22 | -0.22 | 0.584 (0.044) | -0.17 | -0.22 | 0.191 (0.033) | -0.06 | -0.23 | 0.247 (0.043) | 0.00 | 0.00 |
| TSMR |  |  |  |  |  |  |  |  | 0.195 (0.034) | -0.06 | -0.22 | 0.252 (0.045) | 0.00 | 0.00 |
| MVMR | 0.75 | 0 | 0.001 (0.032) | 0.00 | NA | 0.001 (0.03) | 0.00 | NA | 0 (0.005) | 0.00 | NA | -0.048 (9.389) | -0.80 | NA |
| TSMR |  |  |  |  |  |  |  |  | 0 (0.005) | 0.00 | NA | -0.066 (10.008) | -0.82 | NA |
| MVMR |  | 0.2 | 0.239 (0.031) | 0.04 | 0.19 | 0.06 (0.035) | 0.01 | 0.19 | 0.179 (0.021) | 0.03 | 0.19 | 0.762 (0.131) | 0.01 | 0.24 |
| TSMR |  |  |  |  |  |  |  |  | 0.182 (0.022) | 0.03 | 0.21 | 0.773 (0.133) | 0.02 | 0.46 |
| MVMR |  | 0.5 | 0.497 (0.031) | 0.00 | -0.01 | 0.129 (0.057) | 0.00 | 0.03 | 0.368 (0.05) | -0.01 | -0.02 | 0.743 (0.109) | -0.01 | -0.06 |
| TSMR |  |  |  |  |  |  |  |  | 0.372 (0.051) | 0.00 | -0.01 | 0.75 (0.111) | 0.00 | 0.00 |
| MVMR |  | 1 | 0.774 (0.031) | -0.23 | -0.23 | 0.196 (0.103) | -0.05 | -0.22 | 0.579 (0.099) | -0.17 | -0.23 | 0.748 (0.131) | 0.00 | -0.01 |
| TSMR |  |  |  |  |  |  |  |  | 0.582 (0.101) | -0.17 | -0.22 | 0.753 (0.133) | 0.00 | 0.01 |

Total effect = estimated using univariate Mendelian randomisation; direct effect = estimated using multivariable Mendelian randomisation controlling for both exposure and mediator

## sTable 16: Estimated effect sizes and size of bias for simulated effect of a continuous mediator explaining the effect between a continuous exposure and a rare binary outcome using the Mendelian randomisation methods multivariable Mendelian randomisation (MVMR) and two-step Mendelian randomisation (TSMR) on the odds ratio scale (Simulated N=5000)

| **Mediation method** | **True proportion mediated** | **True total effect** | **Total effect (SD)** | **Size of bias (absolute)** | **Size of bias (relative)** | **Direct effect (SD)** | **Size of bias (absolute)** | **Size of bias (relative)** | **Indirect effect (SD)** | **Size of bias (absolute)** | **Size of bias (relative)** | **Proportion mediated (SD)** | **Size of bias (absolute)** | **Size of bias (relative)** |
| --- | --- | --- | --- | --- | --- | --- | --- | --- | --- | --- | --- | --- | --- | --- |
| MVMR | 0 | 1.65 | 1.857 (0.116) | 0.21 | 0.13 | 1.862 (0.115) | 0.21 | 0.13 | -0.005 (0.011) | 0.00 | NA | -0.003 (0.006) | 0.00 | NA |
| TSMR |  |  |  |  |  |  |  |  | -0.001 (0.025) | 0.00 | NA | 0.025 (-0.001) | 0.00 | NA |
| MVMR | 0.05 | 1.00 | 0.999 (0.063) | 0.00 | 0.00 | 0.999 (0.062) | 0.05 | 0.05 | 0 (0.007) | -0.05 | -1.00 | 0 (0.007) | -0.05 | -1.00 |
| TSMR |  |  |  |  |  |  |  |  | 0 (0.029) | -0.05 | -1.01 | 0.029 (-0.001) | -0.05 | -1.02 |
| MVMR |  | 1.22 | 1.361 (0.084) | 0.14 | 0.11 | 1.342 (0.081) | 0.18 | 0.16 | 0.019 (0.01) | -0.04 | -0.69 | 0.014 (0.007) | -0.04 | -0.72 |
| TSMR |  |  |  |  |  |  |  |  | 0.069 (0.028) | 0.01 | 0.13 | 0.028 (0.05) | 0.00 | 0.01 |
| MVMR |  | 1.65 | 1.864 (0.121) | 0.22 | 0.13 | 1.812 (0.117) | 0.25 | 0.16 | 0.052 (0.017) | -0.03 | -0.36 | 0.028 (0.009) | -0.02 | -0.44 |
| TSMR |  |  |  |  |  |  |  |  | 0.161 (0.029) | 0.08 | 0.95 | 0.029 (0.086) | 0.04 | 0.72 |
| MVMR |  | 2.72 | 2.58 (0.168) | -0.14 | -0.05 | 2.467 (0.163) | -0.12 | -0.04 | 0.113 (0.037) | -0.02 | -0.17 | 0.044 (0.014) | -0.01 | -0.12 |
| TSMR |  |  |  |  |  |  |  |  | 0.303 (0.031) | 0.17 | 1.23 | 0.031 (0.118) | 0.07 | 1.36 |
| MVMR | 0.25 | 1.00 | 1.002 (0.063) | 0.00 | 0.00 | 1.002 (0.061) | 0.25 | 0.34 | 0 (0.007) | -0.25 | -1.00 | 0 (0.007) | -0.25 | -1.00 |
| TSMR |  |  |  |  |  |  |  |  | -0.001 (0.029) | -0.25 | -1.00 | 0.029 (-0.002) | -0.25 | -1.01 |
| MVMR |  | 1.22 | 1.359 (0.084) | 0.14 | 0.11 | 1.261 (0.078) | 0.34 | 0.38 | 0.098 (0.022) | -0.21 | -0.68 | 0.072 (0.015) | -0.18 | -0.71 |
| TSMR |  |  |  |  |  |  |  |  | 0.339 (0.034) | 0.03 | 0.11 | 0.034 (0.25) | 0.00 | 0.00 |
| MVMR |  | 1.65 | 1.872 (0.122) | 0.22 | 0.14 | 1.607 (0.117) | 0.37 | 0.30 | 0.265 (0.063) | -0.15 | -0.36 | 0.141 (0.032) | -0.11 | -0.43 |
| TSMR |  |  |  |  |  |  |  |  | 0.803 (0.055) | 0.39 | 0.95 | 0.055 (0.431) | 0.18 | 0.72 |
| MVMR |  | 2.72 | 2.583 (0.173) | -0.14 | -0.05 | 2.055 (0.209) | 0.02 | 0.01 | 0.528 (0.162) | -0.15 | -0.22 | 0.205 (0.061) | -0.05 | -0.18 |
| TSMR |  |  |  |  |  |  |  |  | 1.512 (0.098) | 0.83 | 1.23 | 0.098 (0.588) | 0.34 | 1.35 |
| MVMR | 0.75 | 1.00 | 0.999 (0.065) | 0.00 | 0.00 | 0.999 (0.063) | 0.75 | 3.00 | 0 (0.007) | -0.75 | -1.00 | 0 (0.007) | -0.75 | -1.00 |
| TSMR |  |  |  |  |  |  |  |  | 0 (0.028) | -0.75 | -1.00 | 0.028 (0) | -0.75 | -1.00 |
| MVMR |  | 1.22 | 1.359 (0.086) | 0.14 | 0.11 | 1.083 (0.081) | 0.78 | 2.55 | 0.275 (0.053) | -0.64 | -0.70 | 0.202 (0.036) | -0.55 | -0.73 |
| TSMR |  |  |  |  |  |  |  |  | 1.019 (0.067) | 0.10 | 0.11 | 0.067 (0.752) | 0.00 | 0.00 |
| MVMR |  | 1.65 | 1.865 (0.113) | 0.22 | 0.13 | 1.179 (0.156) | 0.77 | 1.86 | 0.686 (0.143) | -0.55 | -0.44 | 0.368 (0.073) | -0.38 | -0.51 |
| TSMR |  |  |  |  |  |  |  |  | 2.412 (0.151) | 1.18 | 0.95 | 0.151 (1.298) | 0.55 | 0.73 |
| MVMR |  | 2.72 | 2.578 (0.165) | -0.14 | -0.05 | 1.299 (0.304) | 0.62 | 0.91 | 1.28 (0.308) | -0.76 | -0.37 | 0.496 (0.115) | -0.25 | -0.34 |
| TSMR |  |  |  |  |  |  |  |  | 4.545 (0.279) | 2.51 | 1.23 | 0.279 (1.77) | 1.02 | 1.36 |

Total effect = estimated using univariate Mendelian randomisation; direct effect = estimated using multivariable Mendelian randomisation controlling for both exposure and mediator

## sTable 17: Estimated effect sizes and size of bias for simulated effect of a continuous mediator explaining the effect between a continuous exposure and a common binary outcome using the Mendelian randomisation methods multivariable Mendelian randomisation (MVMR) and two-step Mendelian randomisation (TSMR) on the odds ratio scale (Simulated N=5000)

| **Mediation method** | **True proportion mediated** | **True total effect** | **Total effect (SD)** | **Size of bias (absolute)** | **Size of bias (relative)** | **Direct effect (SD)** | **Size of bias (absolute)** | **Size of bias (relative)** | **Indirect effect (SD)** | **Size of bias (absolute)** | **Size of bias (relative)** | **Proportion mediated (SD)** | **Size of bias (absolute)** | **Size of bias (relative)** |
| --- | --- | --- | --- | --- | --- | --- | --- | --- | --- | --- | --- | --- | --- | --- |
| MVMR | 0 | 1.65 | 1.643 (0.052) | -0.01 | 0.00 | 1.65 (0.05) | 0.00 | 0.00 | -0.006 (0.007) | -0.01 | NA | -0.004 (0.004) | 0.00 | NA |
| TSMR |  |  |  |  |  |  |  |  | -0.001 (0.024) | 0.00 | NA | -0.001 (0.015) | 0.00 | NA |
| MVMR | 0.05 | 1.00 | 1 (0.033) | 0.00 | 0.00 | 1 (0.03) | 0.05 | 0.05 | 0 (0.005) | -0.05 | -1.00 | 0 (0.005) | -0.05 | -1.00 |
| TSMR |  |  |  |  |  |  |  |  | 0 (0.027) | -0.05 | -1.01 | -0.001 (0.027) | -0.05 | -1.02 |
| MVMR |  | 1.22 | 1.274 (0.039) | 0.05 | 0.05 | 1.262 (0.036) | 0.10 | 0.09 | 0.012 (0.006) | -0.05 | -0.80 | 0.009 (0.005) | -0.04 | -0.81 |
| TSMR |  |  |  |  |  |  |  |  | 0.064 (0.025) | 0.00 | 0.05 | 0.05 (0.019) | 0.00 | 0.01 |
| MVMR |  | 1.65 | 1.645 (0.049) | 0.00 | 0.00 | 1.611 (0.046) | 0.04 | 0.03 | 0.034 (0.008) | -0.05 | -0.58 | 0.021 (0.005) | -0.03 | -0.58 |
| TSMR |  |  |  |  |  |  |  |  | 0.152 (0.026) | 0.07 | 0.85 | 0.093 (0.015) | 0.04 | 0.85 |
| MVMR |  | 2.72 | 2.171 (0.07) | -0.55 | -0.55 | 2.096 (0.067) | -0.49 | -0.19 | 0.075 (0.013) | -0.06 | -0.45 | 0.034 (0.006) | -0.02 | -0.31 |
| TSMR |  |  |  |  |  |  |  |  | 0.292 (0.024) | 0.16 | 1.15 | 0.134 (0.011) | 0.08 | 1.69 |
| MVMR | 0.25 | 1.00 | 1.001 (0.034) | 0.00 | 0.00 | 1.001 (0.031) | 0.25 | 0.33 | 0 (0.006) | -0.25 | -1.00 | 0 (0.006) | -0.25 | -1.00 |
| TSMR |  |  |  |  |  |  |  |  | -0.001 (0.027) | -0.25 | -1.00 | -0.001 (0.027) | -0.25 | -1.01 |
| MVMR |  | 1.22 | 1.27 (0.038) | 0.05 | 0.05 | 1.199 (0.035) | 0.28 | 0.31 | 0.071 (0.01) | -0.23 | -0.77 | 0.056 (0.007) | -0.19 | -0.78 |
| TSMR |  |  |  |  |  |  |  |  | 0.317 (0.027) | 0.01 | 0.04 | 0.249 (0.019) | 0.00 | 0.00 |
| MVMR |  | 1.65 | 1.646 (0.051) | 0.00 | 0.00 | 1.459 (0.05) | 0.69 | 0.18 | 0.187 (0.025) | -0.07 | -0.55 | 0.113 (0.015) | -0.14 | -0.55 |
| TSMR |  |  |  |  |  |  |  |  | 0.763 (0.032) | 0.51 | 0.85 | 0.464 (0.02) | 0.21 | 0.85 |
| MVMR |  | 2.72 | 2.173 (0.069) | -0.55 | -0.55 | 1.795 (0.08) | 1.01 | -0.12 | 0.377 (0.06) | 0.11 | -0.44 | 0.174 (0.027) | -0.08 | -0.31 |
| TSMR |  |  |  |  |  |  |  |  | 1.463 (0.046) | 1.20 | 1.15 | 0.674 (0.027) | 0.42 | 1.69 |
| MVMR | 0.75 | 1.00 | 1.001 (0.032) | 0.00 | 0.00 | 1.001 (0.03) | 0.75 | 3.01 | 0 (0.005) | -0.75 | -1.00 | 0 (0.005) | -0.75 | -1.00 |
| TSMR |  |  |  |  |  |  |  |  | 0 (0.026) | -0.75 | -1.00 | 0 (0.026) | -0.75 | -1.00 |
| MVMR |  | 1.22 | 1.27 (0.039) | 0.05 | 0.05 | 1.062 (0.038) | 0.76 | 2.48 | 0.208 (0.024) | -0.71 | -0.77 | 0.164 (0.018) | -0.59 | -0.78 |
| TSMR |  |  |  |  |  |  |  |  | 0.956 (0.037) | 0.04 | 0.04 | 0.753 (0.029) | 0.00 | 0.00 |
| MVMR |  | 1.65 | 1.645 (0.051) | 0.00 | 0.00 | 1.14 (0.066) | 0.88 | 1.77 | 0.505 (0.059) | -0.26 | -0.59 | 0.307 (0.035) | -0.44 | -0.59 |
| TSMR |  |  |  |  |  |  |  |  | 2.288 (0.067) | 1.52 | 0.85 | 1.392 (0.053) | 0.64 | 0.86 |
| MVMR |  | 2.72 | 2.170 (0.068) | -0.55 | -0.55 | 1.222 (0.126) | 0.96 | 0.80 | 0.948 (0.126) | 0.16 | -0.54 | 0.437 (0.056) | -0.31 | -0.42 |
| TSMR |  |  |  |  |  |  |  |  | 4.382 (0.12) | 3.59 | 1.15 | 2.021 (0.08) | 1.27 | 1.69 |

Total effect = estimated using univariate Mendelian randomisation; direct effect = estimated using multivariable Mendelian randomisation controlling for both exposure and mediator

|  | **Outcome** | **Mediation method** | **True proportion mediated** | **True total effect** | **Total effect (SD)** | **Size of bias (absolute)** | **Size of bias (relative)** | **Direct effect (SD)** | **Size of bias (absolute)** | **Size of bias (relative)** | **Indirect effect(SD)** | **Size of bias (absolute)** | **Size of bias (relative)** | **Proportion mediated (SD)** | **Size of bias (absolute)** | **Size of bias (relative)** |
| --- | --- | --- | --- | --- | --- | --- | --- | --- | --- | --- | --- | --- | --- | --- | --- | --- |
| **Measurement error in the exposure** | Continuous | Difference | 0.25 | 0.5 | 0.366 (0.009) | 0.17 | 0.33 | 0.078  (0.004) | -0.30 | -0.59 | 0.288 (0.008) | 0.16 | 1.30 | 0.786 (0.011) | 0.54 | 2.14 |
|  |  | Product |  |  |  |  |  |  |  |  | 0.288 (0.008) | 0.16 | 1.30 | 0.786 (0.011) | 0.54 | 0.27 |
|  | Rare binary | Difference |  | 0.025 | 0.021 (0.001) | 0.00 | -0.15 | 0.005 (0.001) | -0.01 | -0.76 | 0.017 (0.001) | 0.01 | 1.66 | 0.787 (0.049) | 0.54 | 2.15 |
|  |  | Product |  |  |  |  |  |  |  |  | 0.017 (0.001) | 0.01 | 1.66 | 0.787 (0.049) | 0.54 | 2.15 |
|  | Common binary | Difference |  | 0.125 | 0.065 (0.002) | -0.06 | -0.48 | 0.014 (0.002) | -0.08 | -0.85 | 0.051 (0.001) | 0.02 | 0.64 | 0.785 (0.027) | 0.54 | 2.14 |
|  |  | Product |  |  |  |  |  |  |  |  | 0.051 (0.001) | 0.02 | 0.64 | 0.785 (0.027) | 0.54 | 2.14 |
| **Measurement error in the mediator** | Continuous | Difference | 0.25 | 0.5 | 1.10 (0.009) | 0.60 | 1.20 | 0.936 (0.01) | 0.56 | 1.12 | 0.164 (0.006) | 0.04 | 0.31 | 0.149 (0.006) | -0.10 | -0.40 |
|  |  | Product |  |  |  |  |  |  |  |  | 0.164 (0.006) | 0.04 | 0.31 | 0.149 (0.006) | -0.10 | -0.05 |
|  | Rare binary | Difference |  | 0.025 | 0.064 (0.001) | 0.04 | 1.54 | 0.054 (0.002) | 0.04 | 1.89 | 0.009 (0.001) | 0.00 | 0.51 | 0.148 (0.021) | -0.10 | -0.41 |
|  |  | Product |  |  |  |  |  |  |  |  | 0.009 (0.001) | 0.00 | 0.51 | 0.148 (0.021) | -0.10 | -0.41 |
|  | Common binary | Difference |  | 0.125 | 0.196 (0.002) | 0.07 | 0.57 | 0.167 (0.004) | 0.07 | 0.78 | 0.029 (0.002) | 0.00 | -0.07 | 0.148 (0.012) | -0.10 | -0.41 |
|  |  | Product |  |  |  |  |  |  |  |  | 0.029 (0.002) | 0.00 | -0.07 | 0.148 (0.012) | -0.10 | -0.41 |

## sTable 18: Estimated effect sizes and size of bias for simulated effect of a continuous mediator explaining the effect between a continuous exposure and continuous outcome (per unit increase in exposure), and a rare binary outcome and common binary outcome using the non-IV difference in coefficients (difference) method and product of coefficients (product) method on the risk or mean difference scale, where measurement error is introduced in either the exposure or mediator (Simulated N=5000)

## sTable 19: Estimated effect sizes and size of bias for simulated effect of a continuous mediator explaining the effect between a continuous exposure and continuous outcome (per unit increase in exposure), and a rare binary outcome and common binary outcome using the Mendelian randomisation methods multivariable Mendelian randomisation (MVMR) and two-step Mendelian randomisation (TSMR) on the risk or mean difference scale, where measurement error is introduced in either the exposure or mediator (Simulated N=5000)

|  | **Outcome** | **Mediation method** | **True proportion mediated** | **True total effect** | **Total effect (SD)** | **Size of bias (absolute)** | **Size of bias (relative)** | **Direct effect (SD)** | **Size of bias (absolute)** | **Size of bias (relative)** | **Indirect effect (SD)** | **Size of bias (absolute)** | **Size of bias (relative)** | **Proportion mediated (SD)** | **Size of bias (absolute)** | **Size of bias (relative)** |
| --- | --- | --- | --- | --- | --- | --- | --- | --- | --- | --- | --- | --- | --- | --- | --- | --- |
| **Measurement error in the exposure** | Continuous | MVMR | 0.25 | 0.5 | 0.499 (0.022) | 0.00 | 0.00 | 0.375 (0.021) | 0.00 | 0.00 | 0.124 (0.013) | 0.00 | -0.01 | 0.249 (0.024) | 0.00 | 0.00 |
|  |  | TSMR |  |  |  |  |  |  |  |  | 0.124 (0.013) | 0.00 | -0.01 | 0.013 (0.249) | 0.00 | 0.00 |
|  | Rare binary | MVMR |  | 0.025 | 0.029 (0.003) | 0.00 | 0.16 | 0.022 (0.003) | 0.00 | 0.16 | 0.007 (0.002) | 0.00 | 0.15 | 0.252 (0.067) | 0.00 | 0.01 |
|  |  | TSMR |  |  |  |  |  |  |  |  | 0.007 (0.002) | 0.00 | 0.15 | 0.002 (0.252) | 0.00 | 0.01 |
|  | Common binary | MVMR |  | 0.125 | 0.089 (0.006) | -0.04 | -0.29 | 0.067 (0.006) | -0.03 | -0.29 | 0.022 (0.003) | -0.01 | -0.29 | 0.248 (0.039) | 0.00 | -0.01 |
|  |  | TSMR |  |  |  |  |  |  |  |  | 0.022 (0.003) | -0.01 | -0.29 | 0.248 (0.039) | 0.00 | -0.01 |
| **Measurement error in the mediator** | Continuous | MVMR | 0.25 | 0.5 | 0.499 (0.017) | 0.00 | 0.00 | 0.374 (0.018) | 0.00 | 0.00 | 0.125 (0.012) | 0.00 | 0.00 | 0.251 (0.022) | 0.00 | 0.00 |
|  |  | TSMR |  |  |  |  |  |  |  |  | 0.125 (0.012) | 0.00 | 0.00 | 0.012 (0.251) | 0.00 | 0.00 |
|  | Rare binary | MVMR |  | 0.025 | 0.029 (0.003) | 0.00 | 0.15 | 0.022 (0.003) | 0.00 | 0.15 | 0.007 (0.002) | 0.00 | 0.16 | 0.255 (0.068) | 0.00 | 0.02 |
|  |  | TSMR |  |  |  |  |  |  |  |  | 0.007 (0.002) | 0.00 | 0.16 | 0.002 (0.255) | 0.00 | 0.02 |
|  | Common binary | MVMR |  | 0.125 | 0.089 (0.005) | -0.04 | -0.29 | 0.067 (0.006) | -0.03 | -0.29 | 0.022 (0.003) | -0.01 | -0.29 | 0.249 (0.04) | 0.00 | 0.00 |
|  |  | TSMR |  |  |  |  |  |  |  |  | 0.022 (0.003) | -0.01 | -0.29 | 0.249 (0.04) | 0.00 | 0.00 |

Total effect = estimated using univariate Mendelian randomisation; direct effect = estimated using multivariable Mendelian randomisation controlling for both exposure and mediator

## sTable 20: Estimated effect sizes and size of bias for simulated effect of a continuous mediator explaining the effect between a continuous exposure and continuous outcome (per unit increase in exposure), using the Mendelian randomisation methods multivariable Mendelian randomisation (MVMR) and two-step Mendelian randomisation (TSMR) on the mean difference scale, where pleiotropy is simulated in the exposure (Simulated N=5000)

| **Mediation method** | **True total** | **True proportion mediated** | **Total effect (SD)** | **Size of bias (absolute)** | **Size of bias (relative)** | **Direct effect (SD)** | **Size of bias (absolute)** | **Size of bias (relative)** | **Indirect effect (SD)** | **Size of bias (absolute)** | **Size of bias (relative)** |
| --- | --- | --- | --- | --- | --- | --- | --- | --- | --- | --- | --- |
| MVMR | 0.2 | 0.05 | 0.4 (0.01) | 0.2 | 1 | 0.39 (0.01) | 0.2 | 1.05 | 0.01 (0.004) | 0 | 0 |
| TSMR |  |  |  |  |  |  |  |  | 0.01 (0.004) | 0 | 0 |
| MVMR |  | 0.25 |  |  |  | 0.35 (0.01) | 0.2 | 1.33 | 0.05 (0.005) | 0 | 0 |
| TSMR |  |  |  |  |  |  |  |  | 0.05 (0.006) | 0 | 0 |
| MVMR |  | 0.75 |  |  |  | 0.25 (0.01) | 0.2 | 4 | 0.15 (0.009) | 0 | 0 |
| TSMR |  |  |  |  |  |  |  |  | 0.15 (0.013) | 0 | 0 |
| MVMR | 0.5 | 0.05 | 0.7 (0.01) | 0.2 | 0.4 | 0.67 (0.01) | 0.2 | 0.42 | 0.03 (0.004) | 0 | 0 |
| TSMR |  |  |  |  |  |  |  |  | 0.03 (0.006) | 0 | 0 |
| MVMR |  | 0.25 |  |  |  | 0.57 (0.01) | 0.2 | 0.53 | 0.13 (0.008) | 0 | 0 |
| TSMR |  |  |  |  |  |  |  |  | 0.13 (0.015) | 0 | 0 |
| MVMR |  | 0.75 |  |  |  | 0.33 (0.01) | 0.2 | 1.6 | 0.37 (0.022) | 0 | 0 |
| TSMR |  |  |  |  |  |  |  |  | 0.37 (0.032) | 0 | 0 |
| MVMR | 1 | 0.05 | 1.2 (0.01) | 0.2 | 0.2 | 1.15 (0.01) | 0.2 | 0.21 | 0.05 (0.005) | 0 | 0 |
| TSMR |  |  |  |  |  |  |  |  | 0.05 (0.01) | 0 | 0 |
| MVMR |  | 0.25 |  |  |  | 0.95 (0.01) | 0.2 | 0.27 | 0.25 (0.014) | 0 | 0 |
| TSMR |  |  |  |  |  |  |  |  | 0.25 (0.036) | 0 | 0 |
| MVMR |  | 0.75 |  |  |  | 0.45 (0.01) | 0.2 | 0.8 | 0.75 (0.044) | 0 | 0 |
| TSMR |  |  |  |  |  |  |  |  | 0.74 (0.075) | -0.01 | -0.01 |

## sTable 21: Estimated effect sizes and size of bias for simulated effect of a continuous mediator explaining the effect between a continuous exposure and continuous outcome (per unit increase in exposure), using the Mendelian randomisation methods multivariable Mendelian randomisation (MVMR) and two-step Mendelian randomisation (TSMR) on the mean difference scale, where pleiotropy is simulated in the mediator (Simulated N=5000)

| **Mediation method** | **True total** | **True proportion mediated** | **Total effect (SD)** | **Size of bias (absolute)** | **Size of bias (relative)** | **Direct effect (SD)** | **Size of bias (absolute)** | **Size of bias (relative)** | **Indirect effect (SD)** | **Size of bias (absolute)** | **Size of bias (relative)** |
| --- | --- | --- | --- | --- | --- | --- | --- | --- | --- | --- | --- |
| MVMR | 0.2 | 0.05 | 0.2 (0.02) | 0 | 0 | 0.18 (0.01) | -0.01 | -0.05 | 0.02 (0.008) | 0.01 | 0.2 |
| TSMR |  |  |  |  |  |  |  |  | 0.02 (0.008) | 0.01 | 7.4 |
| MVMR |  | 0.25 |  |  |  | 0.1 (0.01) | -0.05 | -0.33 | 0.1 (0.009) | 0.05 | 0.2 |
| TSMR |  |  |  |  |  |  |  |  | 0.1 (0.009) | 0.05 | 0.2 |
| MVMR |  | 0.75 |  |  |  | -0.1 (0.01) | -0.15 | -3 | 0.3 (0.012) | 0.15 | 0.2 |
| TSMR |  |  |  |  |  |  |  |  | 0.3 (0.011) | 0.15 | 0.2 |
| MVMR | 0.5 | 0.05 | 0.5 (0.02) | 0 | 0 | 0.45 (0.01) | -0.03 | -0.06 | 0.05 (0.008) | 0.02 | 0.4 |
| TSMR |  |  |  |  |  |  |  |  | 0.05 (0.009) | 0.02 | 0.4 |
| MVMR |  | 0.25 |  |  |  | 0.25 (0.01) | -0.13 | -0.35 | 0.25 (0.011) | 0.12 | 0.48 |
| TSMR |  |  |  |  |  |  |  |  | 0.25 (0.013) | 0.12 | 0.48 |
| MVMR |  | 0.75 |  |  |  | -0.25 (0.01) | -0.38 | -3.04 | 0.75 (0.022) | 0.37 | 0.49 |
| TSMR |  |  |  |  |  |  |  |  | 0.75 (0.015) | 0.37 | 0.49 |
| MVMR | 1 | 0.05 | 1 (0.02) | 0 | 0 | 0.9 (0.01) | -0.05 | -0.05 | 0.1 (0.009) | 0.05 | 1 |
| TSMR |  |  |  |  |  |  |  |  | 0.1 (0.012) | 0.05 | 0 |
| MVMR |  | 0.25 |  |  |  | 0.5 (0.01) | -0.25 | -0.33 | 0.5 (0.016) | 0.25 | 1 |
| TSMR |  |  |  |  |  |  |  |  | 0.5 (0.027) | 0.25 | 1 |
| MVMR |  | 0.75 |  |  |  | -0.5 (0.01) | -0.75 | -3 | 1.5 (0.043) | 0.75 | 1 |
| TSMR |  |  |  |  |  |  |  |  | 1.5 (0.027) | 0.75 | 1 |

## sTable 22: Estimated effect sizes and size of bias for simulated effect of a continuous mediator explaining the effect a continuous exposure and continuous outcome (per unit increase in exposure), and a rare binary outcome and common binary outcome using the Mendelian randomisation methods multivariable Mendelian randomisation (MVMR) and two-step Mendelian randomisation (TSMR) on the risk or mean difference scale, where simulated total effects are imprecise (Simulated N=1000)

| **Outcome** | **Mediation method** | **True total effect** | **True proportion mediated** | **Total effect (SD)** | **Size of bias (absolute)** | **Size of bias (relative)** | **Direct effect (SD)** | **Size of bias (absolute)** | **Size of bias (relative)** | **Indirect effect (SD)** | **Size of bias (absolute)** | **Size of bias (relative)** | **Proportion mediated (SD)** | **Size of bias (absolute)** | **Size of bias (relative)** |
| --- | --- | --- | --- | --- | --- | --- | --- | --- | --- | --- | --- | --- | --- | --- | --- |
| Contin-uous | MVMR | 0.2 | 0.05 | 0.319 (0.058) | 0.00 | 0.00 | 0.334 (0.066) | 0.00 | 0.00 | 0.2 (0.144) | 0.00 | -0.07 | -0.137 (14.419) | -0.19 | -3.73 |
|  | TSMR |  |  |  |  |  |  |  |  | 0.009 (0.015) | 0.00 | -0.07 | -0.137 (14.419) | -0.19 | -3.73 |
|  | MVMR |  | 0.25 | 0.419 (0.076) | -0.01 | -0.03 | 0.439 (0.088) | -0.01 | -0.05 | 0.194 (0.147) | 0.00 | 0.02 | 0.228 (3.527) | -0.02 | -0.09 |
|  | TSMR |  |  |  |  |  |  |  |  | 0.051 (0.03) | 0.00 | 0.02 | 0.228 (3.527) | -0.02 | -0.09 |
|  | MVMR |  | 0.75 | 0.648 (0.113) | 0.00 | 0.00 | 0.679 (0.131) | 0.00 | 0.03 | 0.2 (0.144) | 0.00 | -0.01 | 0.849 (9.156) | 0.10 | 0.13 |
|  | TSMR |  |  |  |  |  |  |  |  | 0.148 (0.076) | 0.00 | -0.01 | 0.849 (9.156) | 0.10 | 0.13 |
| Rare binary | MVMR | 0.01 | 0.05 | 0.01 (0.004) | 0.00 | -0.39 | 0.352 (0.154) | 0.00 | -0.39 | 0.006 (0.009) | 0.00 | -0.38 | 0.006 (0.009) | -0.13 | -2.56 |
|  | TSMR |  |  |  |  |  |  |  |  | 0 (0.001) | 0.00 | -0.38 | -0.078 (2.288) | -0.13 | -2.56 |
|  | MVMR |  | 0.25 | 0.013 (0.005) | 0.00 | -0.43 | 0.456 (0.216) | 0.00 | -0.46 | 0.006 (0.01) | 0.00 | -0.36 | 0.004 (0.01) | -0.14 | -0.55 |
|  | TSMR |  |  |  |  |  |  |  |  | 0.002 (0.002) | 0.00 | -0.36 | 0.113 (2.935) | -0.14 | -0.55 |
|  | MVMR |  | 0.75 | 0.02 (0.008) | 0.00 | -0.39 | 0.702 (0.314) | 0.00 | -0.38 | 0.006 (0.01) | 0.00 | -0.39 | 0.002 (0.011) | -1.48 | -1.97 |
|  | TSMR |  |  |  |  |  |  |  |  | 0.005 (0.005) | 0.00 | -0.39 | -0.731 (42.634) | -1.48 | -1.97 |
| Common binary | MVMR | 0.05 | 0.05 | 0.03 (0.008) | -0.03 | -0.62 | 0.334 (0.098) | -0.03 | -0.62 | 0.019 (0.019) | 0.00 | -0.65 | 0.018 (0.019) | 0.00 | -0.05 |
|  | TSMR |  |  |  |  |  |  |  |  | 0.001 (0.002) | 0.00 | -0.65 | 0.047 (2.153) | 0.00 | -0.05 |
|  | MVMR |  | 0.25 | 0.04 (0.01) | -0.03 | -0.63 | 0.44 (0.124) | -0.02 | -0.64 | 0.018 (0.019) | -0.01 | -0.62 | 0.013 (0.019) | -1.68 | -6.72 |
|  | TSMR |  |  |  |  |  |  |  |  | 0.005 (0.004) | -0.01 | -0.62 | -1.431 (48.68) | -1.68 | -6.72 |
|  | MVMR |  | 0.75 | 0.062 (0.015) | -0.03 | -0.61 | 0.682 (0.191) | -0.01 | -0.57 | 0.019 (0.019) | -0.02 | -0.62 | 0.005 (0.021) | -0.02 | -0.02 |
|  | TSMR |  |  |  |  |  |  |  |  | 0.014 (0.01) | -0.02 | -0.62 | 0.731 (16.786) | -0.02 | -0.02 |

Total effect = estimated using univariate Mendelian randomisation; direct effect = estimated using multivariable Mendelian randomisation controlling for both exposure and mediator

## sTable 23: Estimated effect sizes and size of bias for simulated effect of a continuous mediator explaining the effect between a continuous exposure and continuous outcome using the Mendelian randomisation methods multivariable Mendelian randomisation (MVMR) and two-step Mendelian randomisation (TSMR) where true simulated total effects are small (Simulated N=5000)

| **Mediation method** | **True proportion mediated** | **True total effect** | **Total effect (SD)** | **Size of bias (absolute)** | **Size of bias (relative)** | **Direct effect (SD)** | **Size of bias (absolute)** | **Size of bias (relative)** | **Indirect effect (SD)** | **Size of bias (absolute)** | **Size of bias (relative)** | **Proportion mediated (SD)** | **Size of bias (absolute)** | **Size of bias (relative)** |
| --- | --- | --- | --- | --- | --- | --- | --- | --- | --- | --- | --- | --- | --- | --- |
| MVMR | 0.05 | 0.01 | 0.01 (0.017) | 0.00 | -0.03 | 0.009 (0.014) | 0.00 | -0.02 | 0.00 (0.004) | 0.00 | -0.09 | -0.448 (15.516) | -0.50 | -9.97 |
| TSMR |  |  |  |  |  |  |  |  | 0.00 (0.004) | 0.00 | -0.09 | -0.448 (15.516) | -0.50 | -9.97 |
| MVMR |  | 0.05 | 0.05 (0.017) | 0.00 | 0.01 | 0.048 (0.014) | 0.00 | 0.01 | 0.003 (0.004) | 0.00 | 0.04 | 0.027 (0.113) | -0.02 | -0.46 |
| TSMR |  |  |  |  |  |  |  |  | 0.003 (0.004) | 0.00 | 0.04 | 0.027 (0.113) | -0.02 | -0.46 |
| MVMR |  | 0.1 | 0.1 (0.018) | 0.00 | 0.00 | 0.095 (0.015) | 0.00 | 0.00 | 0.005 (0.004) | 0.00 | -0.02 | 0.044 (0.039) | -0.01 | -0.11 |
| TSMR |  |  |  |  |  |  |  |  | 0.005 (0.004) | 0.00 | -0.02 | 0.044 (0.039) | -0.01 | -0.11 |
| MVMR | 0.25 | 0.01 | 0.01 (0.017) | 0.00 | -0.04 | 0.007 (0.014) | 0.00 | -0.05 | 0.002 (0.004) | 0.00 | -0.02 | 0.198 (5.108) | -0.05 | -0.21 |
| TSMR |  |  |  |  |  |  |  |  | 0.002 (0.004) | 0.00 | -0.02 | 0.198 (5.108) | -0.05 | -0.21 |
| MVMR |  | 0.05 | 0.05 (0.018) | 0.00 | -0.01 | 0.037 (0.014) | 0.00 | -0.01 | 0.012 (0.004) | 0.00 | -0.01 | 0.257 (0.124) | 0.01 | 0.03 |
| TSMR |  |  |  |  |  |  |  |  | 0.012 (0.004) | 0.00 | -0.01 | 0.257 (0.124) | 0.01 | 0.03 |
| MVMR |  | 0.1 | 0.099 (0.017) | 0.00 | -0.01 | 0.074 (0.014) | 0.00 | -0.01 | 0.025 (0.004) | 0.00 | -0.02 | 0.251 (0.033) | 0.00 | 0.00 |
| TSMR |  |  |  |  |  |  |  |  | 0.025 (0.004) | 0.00 | -0.02 | 0.251 (0.033) | 0.00 | 0.00 |
| MVMR | 0.75 | 0.01 | 0.01 (0.017) | 0.00 | -0.02 | 0.002 (0.014) | 0.00 | -0.04 | 0.007 (0.004) | 0.00 | -0.01 | 2.062 (68.799) | 1.31 | 1.75 |
| TSMR |  |  |  |  |  |  |  |  | 0.007 (0.004) | 0.00 | -0.01 | 2.062 (68.799) | 1.31 | 1.75 |
| MVMR |  | 0.05 | 0.051 (0.017) | 0.00 | 0.02 | 0.013 (0.014) | 0.00 | 0.06 | 0.038 (0.005) | 0.00 | 0.00 | 0.901 (1.63) | 0.15 | 0.20 |
| TSMR |  |  |  |  |  |  |  |  | 0.038 (0.005) | 0.00 | 0.00 | 0.901 (1.63) | 0.15 | 0.20 |
| MVMR |  | 0.1 | 0.1 (0.017) | 0.00 | 0.00 | 0.025 (0.015) | 0.00 | 0.00 | 0.075 (0.007) | 0.00 | 0.00 | 0.767 (0.117) | 0.02 | 0.02 |
| TSMR |  |  |  |  |  |  |  |  | 0.075 (0.007) | 0.00 | 0.00 | 0.767 (0.117) | 0.02 | 0.02 |

Total effect = estimated using univariate Mendelian randomisation; direct effect = estimated using multivariable Mendelian randomisation controlling for both exposure and mediator

## sTable 24: Estimated effect sizes and size of bias for simulated effect of a continuous mediator explaining the effect between a continuous exposure and continuous outcome, rare binary outcome and common binary outcome using the non-IV difference in coefficients (difference) method and product of coefficients (product) method on the risk or mean difference scale, where simulated total effects are imprecise (Simulated N=1000)

| **Outcome** | **Mediation method** | **True total effect** | **True proportion mediated** | **Total effect (SD)** | **Size of bias (absolute)** | **Size of bias (relative)** | **Direct effect (SD)** | **Size of bias (absolute)** | **Size of bias (relative)** | **Indirect effect (SD)** | **Size of bias (absolute)** | **Size of bias (relative)** | **Proportion mediated (SD)** | **Size of bias (absolute)** | **Size of bias (relative)** |
| --- | --- | --- | --- | --- | --- | --- | --- | --- | --- | --- | --- | --- | --- | --- | --- |
| Continuous | Difference | 0.2 | 0.05 | 0.961 (0.076) | 0.76 | 3.80 | 0.642 (0.095) | 0.45 | 2.26 | 0.961 (0.076) | 0.13 | 12.86 | 0.642 (0.095) | 0.28 | 5.67 |
|  | Product |  |  |  |  |  |  |  |  | 0.319 (0.058) | 0.13 | 12.86 | 0.334 (0.066) | 0.28 | 0.14 |
|  | Difference |  | 0.25 | 0.962 (0.079) | 0.76 | 3.81 | 0.542 (0.109) | 0.39 | 1.96 | 0.962 (0.079) | 0.27 | 5.38 | 0.542 (0.109) | 0.19 | 0.76 |
|  | Product |  |  |  |  |  |  |  |  | 0.419 (0.076) | 0.27 | 5.38 | 0.439 (0.088) | 0.19 | 0.09 |
|  | Difference |  | 0.75 | 0.961 (0.079) | 0.76 | 3.81 | 0.313 (0.137) | 0.26 | 1.31 | 0.961 (0.079) | 0.60 | 3.99 | 0.313 (0.137) | -0.07 | -0.09 |
|  | Product |  |  |  |  |  |  |  |  | 0.648 (0.113) | 0.60 | 3.99 | 0.679 (0.131) | -0.07 | -0.04 |
| Rare binary | Difference | 0.01 | 0.05 | 0.03 (0.005) | 0.02 | 1.98 | 0.02 (0.006) | 0.01 | 1.07 | 0.03 (0.005) | 0.00 | 1.31 | 0.02 (0.006) | 0.30 | 6.04 |
|  | Product |  |  |  |  |  |  |  |  | 0.01 (0.004) | 0.00 | 1.31 | 0.352 (0.154) | 0.30 | 6.04 |
|  | Difference |  | 0.25 | 0.03 (0.005) | 0.02 | 1.97 | 0.017 (0.008) | 0.01 | 1.22 | 0.03 (0.005) | 0.01 | 2.22 | 0.017 (0.008) | 0.21 | 0.82 |
|  | Product |  |  |  |  |  |  |  |  | 0.013 (0.005) | 0.01 | 2.22 | 0.456 (0.216) | 0.21 | 0.82 |
|  | Difference |  | 0.75 | 0.03 (0.005) | 0.02 | 2.00 | 0.01 (0.01) | 0.01 | 2.88 | 0.03 (0.005) | 0.02 | 2.37 | 0.01 (0.01) | -0.05 | -0.06 |
|  | Product |  |  |  |  |  |  |  |  | 0.02 (0.008) | 0.02 | 2.37 | 0.702 (0.314) | -0.05 | -0.06 |
| Common binary | Difference | 0.05 | 0.05 | 0.092 (0.01) | 0.04 | 0.84 | 0.062 (0.013) | 0.01 | 0.30 | 0.092 (0.01) | -0.02 | -6.86 | 0.062 (0.013) | 0.28 | 5.69 |
|  | Product |  |  |  |  |  |  |  |  | 0.03 (0.008) | -0.02 | -6.86 | 0.334 (0.098) | 0.28 | 5.69 |
|  | Difference |  | 0.25 | 0.092 (0.009) | 0.04 | 0.84 | 0.052 (0.014) | 0.01 | 0.39 | 0.092 (0.009) | 0.00 | 0.20 | 0.052 (0.014) | 0.19 | 0.76 |
|  | Product |  |  |  |  |  |  |  |  | 0.04 (0.01) | 0.00 | 0.20 | 0.44 (0.124) | 0.19 | 0.76 |
|  | Difference |  | 0.75 | 0.092 (0.01) | 0.04 | 0.84 | 0.03 (0.019) | 0.02 | 1.41 | 0.092 (0.01) | 0.05 | 1.31 | 0.03 (0.019) | -0.07 | -0.09 |
|  | Product |  |  |  |  |  |  |  |  | 0.062 (0.015) | 0.05 | 1.31 | 0.682 (0.191) | -0.07 | -0.09 |

## sTable 25: Estimated effect sizes and size of bias for simulated effect of a continuous mediator explaining the effect between a continuous exposure and continuous outcome using the non-IV difference in coefficients (difference) method and product of coefficients (product) method on the mean difference scale where true total effects simulated are small (Simulated N=5000)

| **Mediation method** | **True total effect** | **True proportion mediated** | **Total effect (SD)** | **Size of bias (absolute)** | **Size of bias (relative)** | **Direct effect (SD)** | **Size of bias (absolute)** | **Size of bias (relative)** | **Indirect effect (SD)** | **Size of bias (absolute)** | **Size of bias (relative)** | **Proportion mediated (SD)** | **Size of bias (absolute)** | **Size of bias (relative)** |
| --- | --- | --- | --- | --- | --- | --- | --- | --- | --- | --- | --- | --- | --- | --- |
| Difference | 0.05 | 0.01 | 0.61 (0.009) | 0.60 | 60.05 | 0.342 (0.007) | 0.33 | 33.25 | 0.268 (0.007) | 0.26 | 517.96 | 0.44 (0.009) | 0.39 | 7.80 |
| Product |  |  |  |  |  |  |  |  | 0.268 (0.007) | 0.26 | 517.96 | 0.44 (0.009) | 0.39 | 0.19 |
| Difference |  | 0.05 | 0.65 (0.009) | 0.60 | 12.00 | 0.377 (0.007) | 0.33 | 6.58 | 0.273 (0.007) | 0.23 | 90.32 | 0.42 (0.009) | 0.37 | 7.41 |
| Product |  |  |  |  |  |  |  |  | 0.273 (0.007) | 0.23 | 90.32 | 0.42 (0.009) | 0.37 | 0.19 |
| Difference |  | 0.1 | 0.7 (0.009) | 0.60 | 6.00 | 0.42 (0.007) | 0.33 | 3.25 | 0.28 (0.008) | 0.18 | 36.95 | 0.4 (0.008) | 0.35 | 6.99 |
| Product |  |  |  |  |  |  |  |  | 0.28 (0.008) | 0.18 | 36.95 | 0.4 (0.008) | 0.35 | 0.17 |
| Difference | 0.25 | 0.01 | 0.61 (0.009) | 0.60 | 59.95 | 0.336 (0.006) | 0.33 | 32.87 | 0.273 (0.007) | 0.27 | 106.31 | 0.448 (0.009) | 0.20 | 0.79 |
| Product |  |  |  |  |  |  |  |  | 0.273 (0.007) | 0.27 | 106.31 | 0.448 (0.009) | 0.20 | 0.10 |
| Difference |  | 0.05 | 0.65 (0.008) | 0.60 | 12.01 | 0.35 (0.007) | 0.31 | 6.26 | 0.3 (0.007) | 0.26 | 21.01 | 0.461 (0.009) | 0.21 | 0.85 |
| Product |  |  |  |  |  |  |  |  | 0.3 (0.007) | 0.26 | 21.01 | 0.461 (0.009) | 0.21 | 0.11 |
| Difference |  | 0.1 | 0.7 (0.009) | 0.60 | 6.00 | 0.367 (0.007) | 0.29 | 2.92 | 0.333 (0.008) | 0.26 | 10.31 | 0.476 (0.008) | 0.23 | 0.90 |
| Product |  |  |  |  |  |  |  |  | 0.333 (0.008) | 0.26 | 10.31 | 0.476 (0.008) | 0.23 | 0.11 |
| Difference | 0.75 | 0.01 | 0.61 (0.009) | 0.60 | 59.99 | 0.323 (0.007) | 0.32 | 32.07 | 0.287 (0.008) | 0.28 | 37.90 | 0.47 (0.009) | -0.28 | -0.37 |
| Product |  |  |  |  |  |  |  |  | 0.287 (0.008) | 0.28 | 37.90 | 0.47 (0.009) | -0.28 | -0.14 |
| Difference |  | 0.05 | 0.65 (0.009) | 0.60 | 12.00 | 0.284 (0.007) | 0.27 | 5.42 | 0.366 (0.008) | 0.35 | 9.44 | 0.564 (0.01) | -0.19 | -0.25 |
| Product |  |  |  |  |  |  |  |  | 0.366 (0.008) | 0.35 | 9.44 | 0.564 (0.01) | -0.19 | -0.09 |
| Difference |  | 0.1 | 0.7 (0.009) | 0.60 | 6.00 | 0.233 (0.008) | 0.21 | 2.08 | 0.467 (0.009) | 0.44 | 5.89 | 0.667 (0.01) | -0.08 | -0.11 |
| Product |  |  |  |  |  |  |  |  | 0.467 (0.009) | 0.44 | 5.89 | 0.667 (0.01) | -0.08 | -0.04 |

## sTable 26: Estimated total effect and direct effect of the exposure on a continuous outcome mediated by a continuous mediator, where an interaction between the exposure and mediator is present using the non-IV difference in coefficients (difference) method and multivariable Mendelian randomisation (MVMR) method (Simulated N = 5000)

| **Mediation method** | **True direct effect** | **True interaction effect** | **Total effect (SD)** | **Direct effect of exposure (SD)** | **Size of bias (absolute)** | **Size of bias (relative)** |
| --- | --- | --- | --- | --- | --- | --- |
| Difference | 0.4 | 0.05 | 0.70 (0.01) | 0.94 (0.01) | 0.54 | 1.35 |
| MVMR |  |  | 0.79 (0.02) | 0.67 (0.01) | 0.27 | 0.68 |
| Difference |  | 0.1 | 0.70 (0.02) | 1.21 (0.01) | 0.81 | 2.03 |
| MVMR |  |  | 1.08 (0.03) | 0.94 (0.02) | 0.54 | 1.35 |
| Difference |  | 0.2 | 0.70 (0.02) | 1.75 (0.01) | 1.35 | 3.38 |
| MVMR |  |  | 1.66 (0.03) | 1.48 (0.02) | 1.08 | 2.7 |

## sTable 27: Estimated indirect effect and proportion mediated by multiple continuous mediators explaining the association between a continuous exposure and continuous outcome in simulation analyses using non-IV methods and Mendelian randomisation methods (Simulated N = 5000)

|  | | Total Effect (true value = 0.45) | Direct Effect (true value = 0.20) | **Mutually adjusting for all mediators**  **(Difference in coefficients/MVMR)** | | | | | | | **Considering each mediator independently**  **(Product of coefficients/TSMR)** | | | | | | |
| --- | --- | --- | --- | --- | --- | --- | --- | --- | --- | --- | --- | --- | --- | --- | --- | --- | --- |
|  |  |  |  | M1 | | M2 | | M3 | | Proportion mediated combined (true value = 0.56) | M1 | | M2 | | M3 | | Proportion mediated combined (true value = 0.56 |
|  |  |  |  | Indirect effect | Proportion mediated | Indirect effect | Proportion mediated | Indirect effect | Proportion mediated |  | Indirect effect | Proportion mediated | Indirect effect | Proportion mediated | Indirect effect | Proportion mediated |  |
| **Non-IV** | Independent mediators | 1.55 (0.02) | 0.26 (0.01) | 0.42 (0.01) | 0.28 (0.01) | 0.42 (0.01) | 0.30 (0.01) | 0.45 (0.01) | 0.35 (0.01) | 0.83 (0.02) | 0.93 (0.02) | 0.60 (0.01) | 1.04 (0.02) | 0.67 (0.01) | 1.21 (0.02) | 0.78 (0.01) | 2.05 |
|  | Related mediators | 1.55 (0.02) | 0.26 (0.01) | 0.42 (0.01) | 0.28 (0.01) | 0.25 (0.01) | 0.16 (0.01) | 0.63 (0.02) | 0.48 (0.01) | 0.83 (0.02) | 0.94 (0.02) | 0.60 (0.01) | 1.04 (0.02) | 0.67 (0.01) | 1.37 (0.02) | 0.88 (0.01) | 2.15 |
| **MR** | Independent mediators | 0.45 (0.03) | 0.20 (0.02) | 0.05 (0.01) | 0.11 (0.02) | 0.08 (0.01) | 0.18 (0.01) | 0.12 (0.01) | 0.27 (0.02) | 0.55 (0.02) | 0.05 (0.01) | 0.11 (0.02) | 0.12 (0.01) | 0.18 (0.01) | 0.12 (0.01) | 0.27 (0.01) | 0.56 |
|  | Related mediators | 0.45 (0.03) | 0.20 (0.02) | 0.05 (0.01) | 0.11 (0.02) | 0.05 (0.01) | 0.11 (0.01) | 0.15 (0.01) | 0.33 (0.02) | 0.55 (0.02) | 0.05 (0.01) | 0.11 (0.02) | 0.08 (0.01) | 0.18 (0.01) | 0.15 (0.02) | 0.33 (0.04) | 0.62 |

True indirect effect of independent mediators: M1 = 0.05; M2 = 0.08; M3 = 0.12

True indirect effect of related mediators: M1 = 0.05; M2 = 0.05; M3 = 0.12; M2 via M3; 0.03

MVMR = multivariable Mendelian randomisation; TSMR = two-step Mendelian randomisation

# Supplementary Figures

## sFigure 1: Directed acyclic graph illustrating Mendelian randomisation and the instrumental variable assumptions required for valid inference


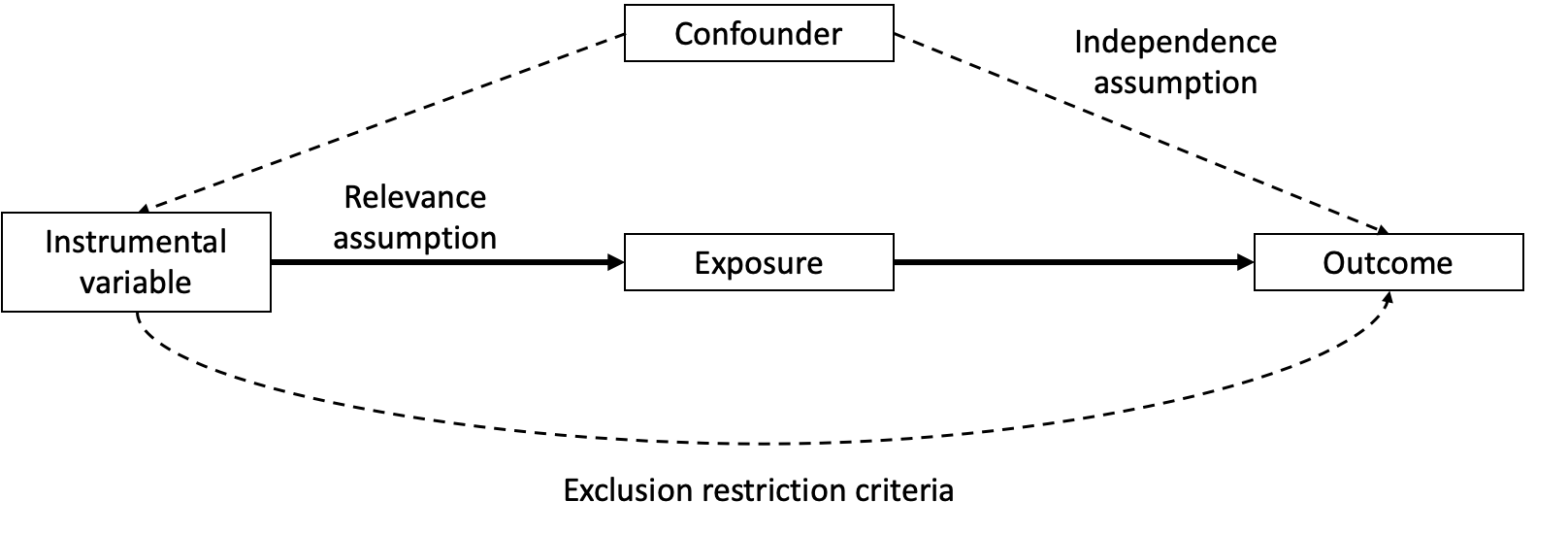


## sFigure 2: Directed acyclic graphs depicting simulation scenarios considering the role of multiple mediators where in A) all three mediators are independent and in B) there is covariance between two of the three mediators


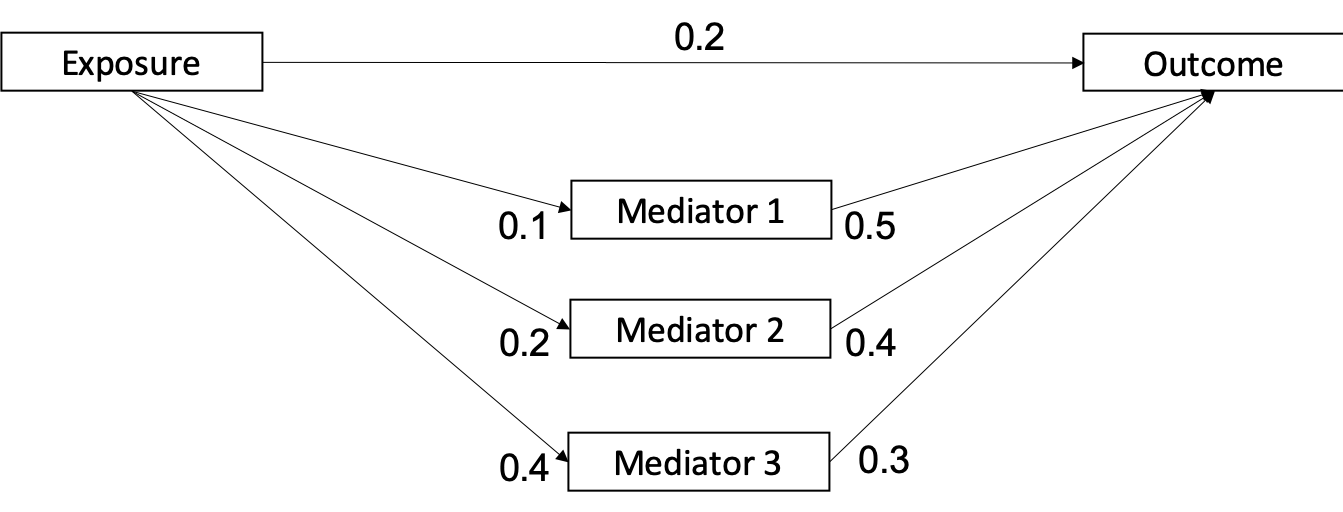


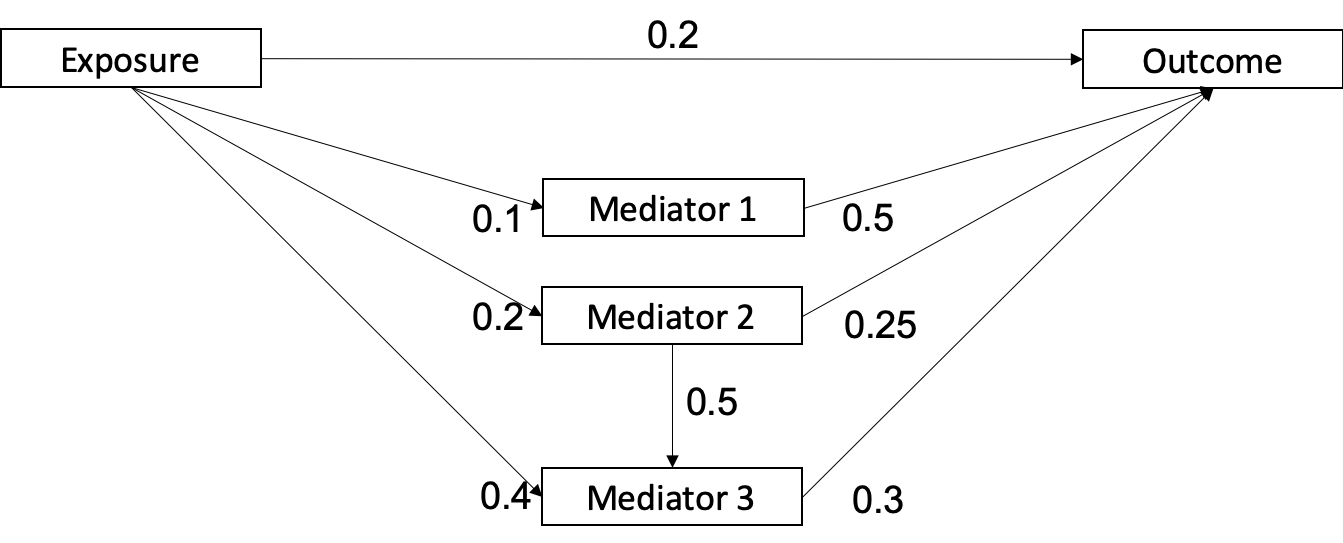


## sFigure 3: Directed acyclic graphs depicting how collider bias can be introduced in non-IV mediation analysis when conditioning on a mediator in the presence of un- or mis- measured mediator-outcome confounders


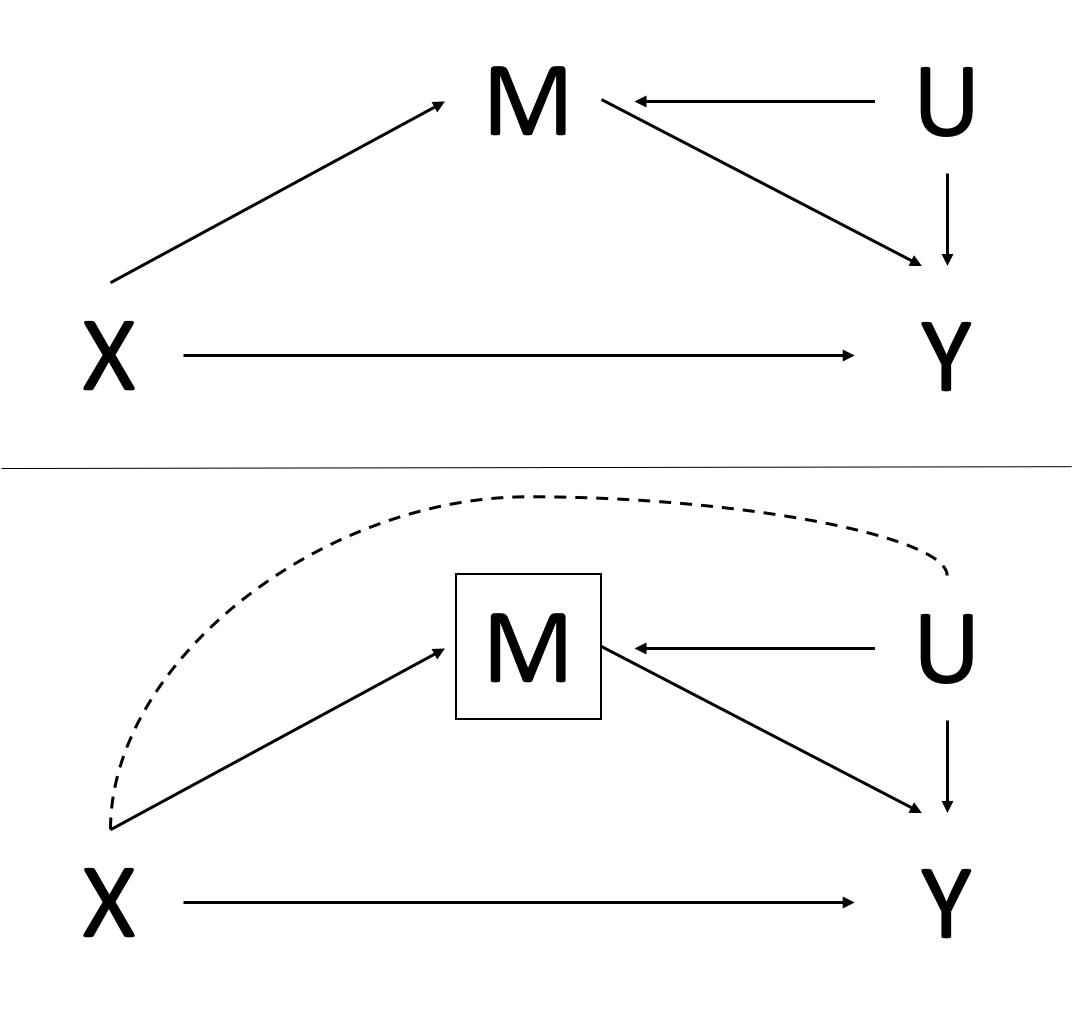


## sFigure 4: Estimates of the proportion mediated and size of absolute bias when weak instrument bias is simulated in A) the exposure and B) the mediator for a true proportion mediated of 0.25 (solid line) (simulated N = 5000)

**A**

**B**


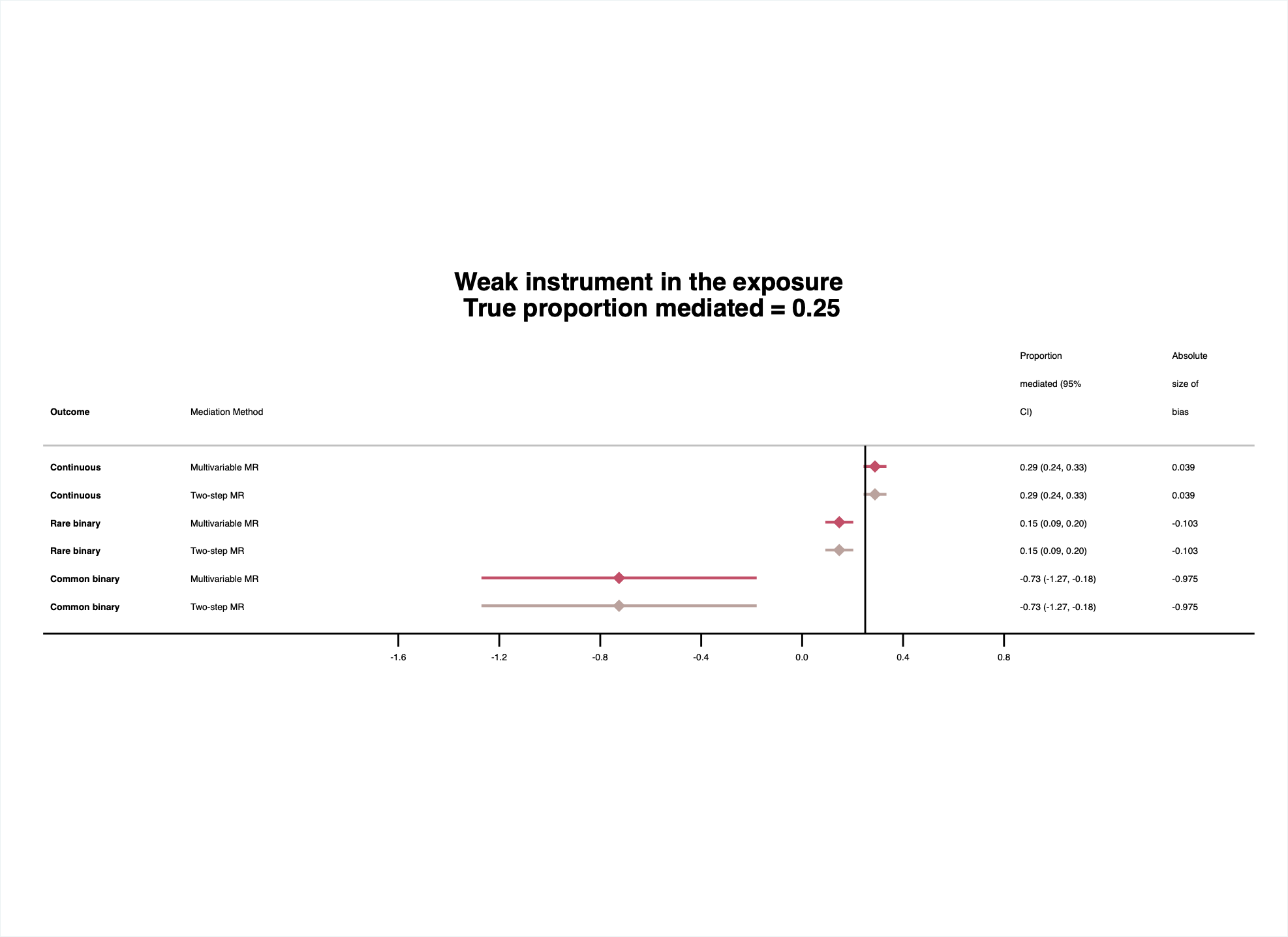

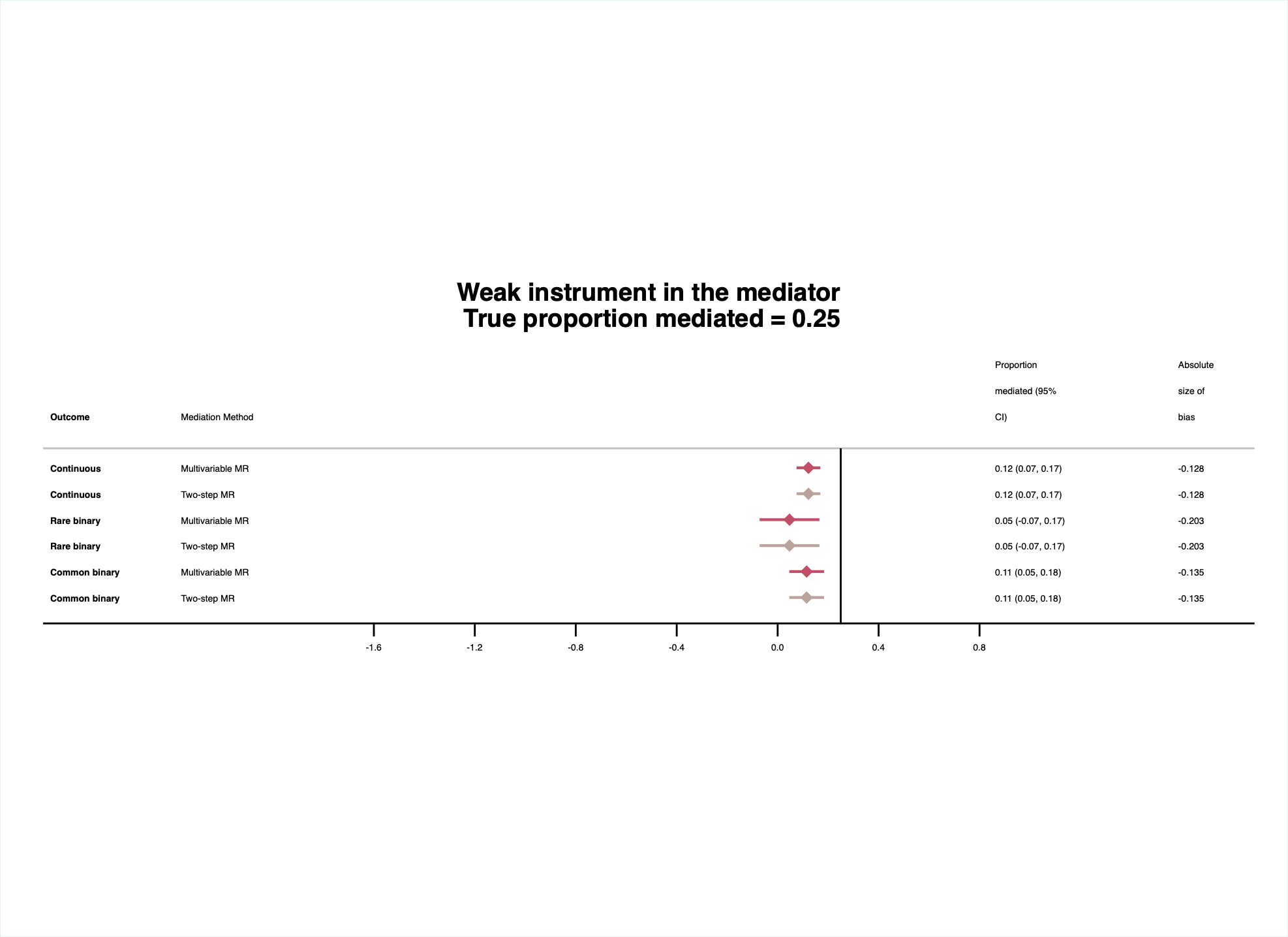


Size of absolute bias

Size of absolute bias

# References

1. VanderWeele TJa. Explanation in causal inference : methods for mediation and interaction. New York, NY : Oxford University Press, [2015]; 2015.
